# Tyro3 facilitates cytoplasmic delivery of extracellular vesicle contents and antigen presentation via major histocompatibility complex class I in dendritic cells

**DOI:** 10.1371/journal.pone.0355054

**Published:** 2026-08-04

**Authors:** Takashi Koyama, Katsuhiko Kojima, Koki Maeda, Takahide Matsui, Hitomi Kosai, Noriko Ishida, Eiji Morita, Mitsunori Fukuda, Nobuyuki Tanaka

**Affiliations:** 1 Division of Tumor Immunobiology, Miyagi Cancer Center Research Institute, Natori, Japan; 2 Division of Tumor Immunobiology, Tohoku University Graduate School of Medicine, Sendai, Japan; 3 Department of Immunology and Microbiology, Shinshu University School of Medicine, Matsumoto, Japan; 4 Department of Biochemistry and Molecular Biology, Faculty of Agriculture and Life Science, Hirosaki University, Hirosaki, Japan; 5 Department of Microbiology and Immunology, Hirosaki University Graduate School of Medicine, Hirosaki, Japan; 6 Laboratory of Membrane Trafficking Mechanisms, Department of Integrative Life Sciences, Graduate School of Life Sciences, Tohoku University, Sendai, Japan; 7 Division of Cancer Molecular Biology, Tohoku University Graduate School of Medicine, Sendai, Japan; 8 Department of Biobank, Tohoku Medical Megabank Organization, Tohoku University, Sendai, Japan; Rutgers University, UNITED STATES OF AMERICA

## Abstract

Extracellular vesicles (EVs) are membrane-bound particles that mediate intercellular communication and are being explored as carriers for antigen delivery in cancer immunotherapy. However, the molecular mechanisms underlying the uptake of antigen-carrying EVs by dendritic cells (DCs) remain unclear. EV uptake is influenced by the recognition of EV surface components by receptors on recipient cells. Phospholipids, including phosphatidylserine (PS), are common structural components of EV membrane. We therefore focused on the TAM receptor tyrosine kinases Tyro3, Axl, and Mertk, which are expressed in antigen-presenting cells and engage PS-exposing membranes through the bridging ligands Growth arrest-specific 6 (Gas6) and Protein S (Pros1). Using the DC2.4 dendritic cell line as a model system, we investigated the involvement of TAM receptors in the uptake of small EVs (sEVs) and subsequent antigen presentation. We found that Tyro3 binding to sEVs was enhanced by Gas6 and Pros1, and that Tyro3 also associated with phosphatidylserine (PS) and phosphatidylglycerol (PG) through these bridging ligands. Among TAM receptors, only Tyro3 markedly enhanced sEV internalization in DC2.4 cells. Deletion analysis indicated that the immunoglobulin C2-type domain (IG2c domain) of Tyro3 is required for efficient sEV association and uptake. Moreover, Tyro3-mediated internalization enabled cross-presentation of sEV-delivered antigens and activation of CD8^+^ T cells. This function required a 46-amino acid intracellular region of Tyro3, which we designated the Tyro3 antigen presentation-related domain (TAPD); deletion of TAPD impaired cross-presentation of sEV-delivered antigens. Finally, we observed an increase in the frequency of Tyro3 ⁺ DCs in the spleens of tumor-bearing mice. These findings suggest that Tyro3 mediates sEV uptake and antigen cross-presentation in DCs and may represent a candidate molecular target for EV-based cancer immunotherapy.

## Introduction

Extracellular vesicles (EVs) play a critical role in intercellular communication. They transport a diverse range of intracellular components, including nucleic acids, proteins, lipids, amino acids, and metabolites [1,2]. In addition, they contribute to the delivery of tumor antigens to antigen-presenting cells (APCs) [3–5]. EVs possess properties such as low immunogenicity, biocompatibility, and an intrinsic ability to interact with target cells. These characteristics make EVs highly advantageous carriers for drug delivery systems (DDS) [6–8]. In cancer immunotherapy, DDS approaches using EVs that specifically target dendritic cells (DCs) are actively being investigated [9–12]. However, the molecular mechanisms governing the uptake of EVs by DCs remain largely unclear.

Recipient cells internalize EVs through various endocytic pathways involving both receptor-mediated and receptor-independent mechanisms [13]. In the receptor-mediated pathway, several cell surface molecules, including lectin family molecules, adhesion molecules, and “eat-me” signal molecules, directly bind to EVs and function as their receptors. Additionally, surface molecules on EVs play a crucial role in their recognition and capture by recipient cells.

The membranes of EVs contain a lipid bilayer, including phosphatidylserine (PS), a phospholipid that is typically abundant on the inner leaflet of the lipid bilayer. EVs expose PS on their outer surface, unlike living cells. Tim-1 and Tim-4, members of the T cell immunoglobulin and mucin domain (TIM) family, have been reported to mediate the internalization of EVs by directly binding to PS [14,15]. However, some other receptors recognize PS indirectly via bridging ligands. The TAM receptors (Tyro3, Axl, and Mertk), which are receptor tyrosine kinases, bind to PS via Growth arrest-specific 6 (Gas6) or Protein S (Pros1) ligands. TAM receptors are generally known as negative regulators of immune responses [16]. However, several studies have reported that they can also promote immune activation in a context-dependent manner [17]. DCs and macrophages express TAM receptors, which function in the phagocytosis of apoptotic cells [18]. While previous reports suggest that TAM also binds to small EVs (sEVs), whether they mediate sEV uptake by DCs and macrophages remains unknown [19,20].

DCs play a central role in activating CD8⁺ and CD4 ⁺ T cells through antigen presentation, serving as key players in anti-microbial and anti-tumor immunity. Effective induction of antitumor immunity requires activation of CD8 ⁺ T cells via antigen presentation by DCs. To achieve this via EV-based antigen delivery, robust uptake of EVs by DCs is essential. Therefore, understanding the mechanisms underlying sEV uptake by DCs is critical for the development of EV-based DDS strategies. In this study, we hypothesized and tested whether TAM receptors in DCs mediate the capture and uptake of sEVs, aiming to provide mechanistic insights relevant to the development of EV-based antigen delivery systems.

## Results

### Gas6 and Pros1 differentially facilitate sEV binding to individual TAM receptor extracellular domains

**Fig 1:**
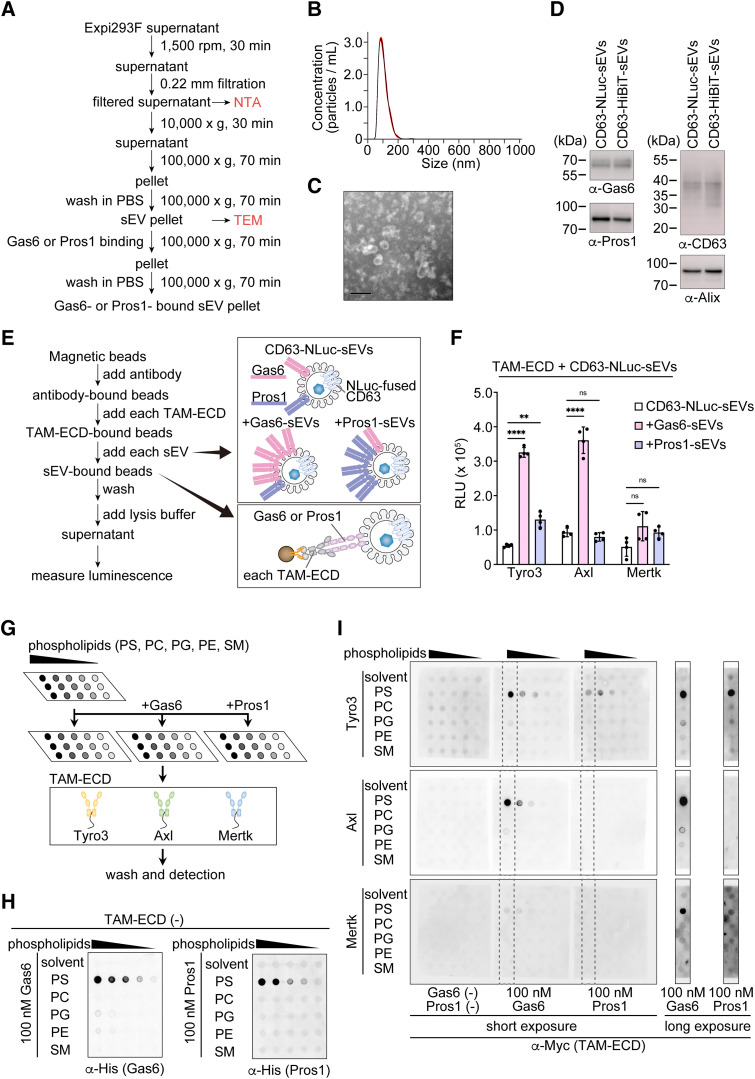
Effects of Gas6 and Pros1 on the binding of individual TAM-ECDs to sEVs and phospholipids. (A) Preparation workflow for sEVs used in this study. Artificially labeled sEVs were isolated from the conditioned medium of Expi293F cells by differential ultracentrifugation (dUC). Exogenous Gas6 and Pros1 were added separately to CD63-NLuc-sEVs, and unbound proteins were then removed by ultracentrifugation. (B) The size distribution of CD63-NLuc-sEVs in the filtered supernatant before dUC was analyzed by nanoparticle tracking analysis (NTA). (C) Representative transmission electron microscopy (TEM) image of CD63-NLuc-sEVs purified by dUC and negatively stained with TI-blue. Scale bar, 200 nm. (D) Intrinsic Gas6 and Pros1 in CD63-NLuc-sEV and CD63-HiBiT-sEV pellets were detected by western blotting. CD63 and Alix were used as sEV markers. (E) Schematic diagram of the pull-down assay. (F) Binding of CD63-NLuc-sEVs, + Gas6-sEVs, or +Pros1-sEVs to each TAM-ECD was quantified by measuring NanoLuc luciferase activity. Data are presented as mean ± SD (n = 4). (G) Schematic diagram of the phospholipid overlay assay. Membranes spotted with phospholipids were incubated with or without TAM ligand, followed by incubation with each TAM-ECD. (H, I) Direct binding of Gas6 or Pros1 to phospholipids (H) and indirect binding of individual TAM-ECDs to phospholipids via Gas6 or Pros1 (I) were detected by western blotting. Longer-exposure images of the area indicated by the gray dotted line are shown on the right. In panels F–I, Tyro3-ECD, Axl-ECD, and Mertk-ECD are denoted as Tyro3, Axl, and Mertk, respectively.

We first examined whether the extracellular domain (ECD) of each TAM receptor interacts with sEVs. To this end, we performed a binding assay using recombinant TAM receptor ECD proteins and CD63-NLuc-sEVs. CD63-NLuc-sEVs carry NanoLuc luciferase (NLuc)-fused CD63, a representative EV-associated marker protein. A schematic overview of the sEV isolation procedure is shown in Fig 1A. To generate artificially labeled sEVs, Expi293F cells were transfected with expression plasmids encoding CD63-based fusion proteins. The culture supernatant was filtered to retain only the sEV fraction. The filtered culture supernatant containing CD63-NLuc-sEVs was analyzed by nanoparticle tracking analysis (NTA). The resulting particle size distribution trace is shown (Fig 1B). The particles in the supernatant exhibited an average size of approximately 105 nm and a mode size of approximately 88 nm, indicating a relatively narrow size distribution characteristic of typical sEVs [2]. Differential ultracentrifugation (dUC) was used to isolate sEVs. Negative-stain TEM analysis revealed particles with diameters of approximately 100 nm in the isolated fraction (Fig 1C). The observed particle size was consistent with the NTA results. Previous studies have shown that PS-positive exosomes can activate TAM receptors through Gas6 or Pros1 [20]. This finding suggests that Gas6 or Pros1 may bridge sEVs and TAM receptors. Therefore, we examined whether intrinsic Gas6 and Pros1 were present in our sEV preparations (Fig 1D). Western blot analysis detected both Gas6 and Pros1 in the CD63-NLuc-sEV fractions, together with the sEV-associated proteins CD63 and Alix. To assess the role of these ligands in interactions between TAM receptors and sEVs, we prepared ligand-opsonized CD63-NLuc-sEVs by preincubating CD63-NLuc-sEVs with recombinant Gas6 or Pros1. These preparations were referred to as +Gas6-sEVs and +Pros1-sEVs, respectively. We performed pull-down assays using CD63-NLuc-sEVs, + Gas6-sEVs, and +Pros1-sEVs in combination with Tyro3-ECD, Axl-ECD, and Mertk-ECD (Fig 1E). In this assay, NLuc activity from NLuc-labeled sEV preparations was used as a readout for sEV binding to individual TAM-ECDs. Therefore, this assay was designed to compare the relative binding of CD63-NLuc-labeled sEV preparations to each TAM-ECD under different ligand conditions using equivalent NLuc inputs. The amount of sEVs bound to each TAM-ECD was quantified by measuring NLuc-dependent luciferase activity in the pull-down fractions (Fig 1F). When CD63-NLuc-sEVs were incubated with each TAM-ECD without additional Gas6 and Pros1 ligands, we detected 5.5 × 10^4^ to 1.0 × 10^5^ RLU of NLuc activity. When Tyro3-ECD was incubated with +Gas6-sEVs or +Pros1-sEVs, NLuc activity was approximately 6-fold higher with +Gas6-sEVs and 2-fold higher with +Pros1-sEVs than with CD63-NLuc-sEVs. When Axl-ECD was incubated with +Gas6-sEVs, NLuc activity was approximately 4-fold higher than that detected with CD63-NLuc-sEVs, whereas no increase was observed with +Pros1-sEVs. However, in the case of Mertk-ECD, neither +Gas6-sEVs nor +Pros1-sEVs resulted in a significant increase in NLuc activity compared to CD63-NLuc-sEVs. These results indicate that Tyro3-ECD preferentially binds to sEVs opsonized with either Gas6 or Pros1, while Axl-ECD exhibits selective binding mainly to Gas6-opsonized sEVs. In contrast, Mertk-ECD shows limited interaction with sEVs, regardless of opsonization. Thus, bridging molecules such as Gas6 and Pros1 facilitate the binding of sEVs to Tyro3-ECD and Axl-ECD under these conditions.

### Gas6 and Pros1 mediate the indirect binding of individual TAM-ECDs to phospholipids

TAM receptors bind to PS through the bridging molecules Gas6 and Pros1 [21]. As various phospholipids are present on the surface of sEVs, we examined the interaction between each TAM-ECD and five phospholipid species reported to be present on sEV membranes (Fig 1G) [22,23]. We assessed the direct binding of Gas6 and Pros1 to each phospholipid (Fig 1H). Pros1 bound selectively to PS, while Gas6 showed strong binding to PS and weaker binding to phosphatidylglycerols (PG) and phosphatidylethanolamines (PE). We next examined whether Gas6 or Pros1 mediates the association between individual TAM-ECDs and phospholipids. (Fig 1I). In the absence of these ligands, none of the three TAM-ECDs showed detectable binding to phospholipids. In the presence of Gas6, binding of the three TAM-ECDs to PS was detected. Tyro3-ECD and Axl-ECD exhibited weak interactions with PG and PE, whereas Mertk-ECD showed only minimal binding to PS. In the presence of Pros1, we found only Tyro3-ECD binding to PS. Thus, Tyro3-ECD can bind to phospholipids via both Gas6 and Pros1, while Axl-ECD binds preferentially via Gas6. In contrast, Mertk-ECD demonstrates limited binding capacity under these conditions.

### Tyro3 on recipient cells facilitates internalization of sEVs

**Fig 2:**
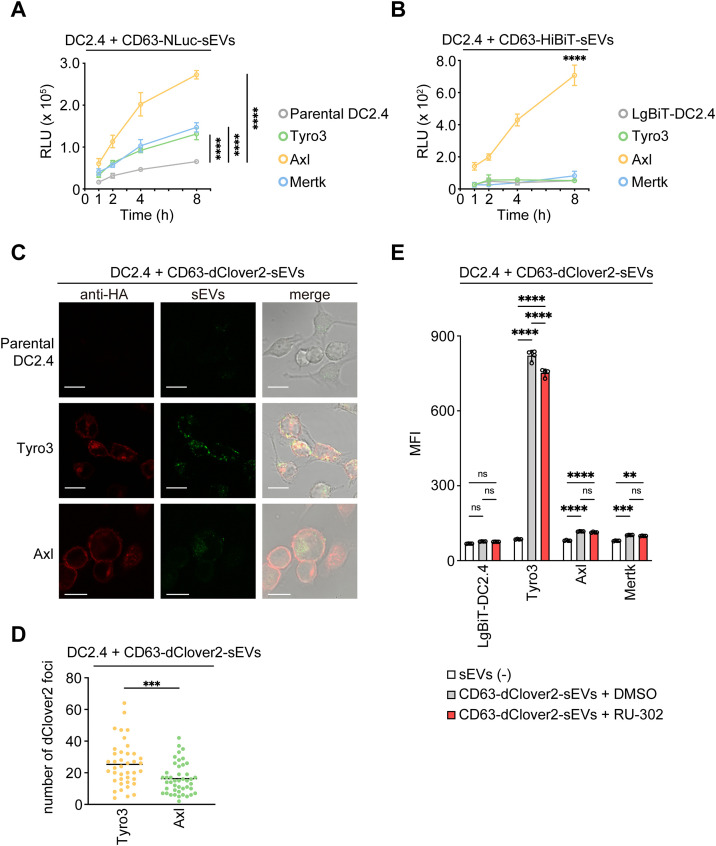
Tyro3 enhances the binding and internalization of sEVs in DC2.4 cells. (A) Association of CD63-NLuc-sEVs with parental and TAM receptor-expressing DC2.4 cells. Cells were incubated with CD63-NLuc-sEVs for the indicated time periods, and cell-associated sEVs were quantified by measuring NanoLuc luciferase activity. Data are presented as mean ± SD (n = 4). (B) Uptake of CD63-HiBiT-sEVs by LgBiT-DC2.4 cells and LgBiT-DC2.4 cells stably expressing Tyro3, Axl, or Mertk. Cells were incubated with CD63-HiBiT-sEVs for the indicated time periods, and HiBiT–LgBiT complementation was quantified by measuring NanoLuc luciferase activity. Data are presented as mean ± SD (n = 4). (C) Confocal microscopy images of CD63-dClover2-sEV uptake by parental and TAM receptor-expressing DC2.4 cells. Cells were stained with an anti-HA antibody to detect HA-tagged TAM receptors (red). CD63-dClover2-sEVs are shown in green, and merged images are shown in the right panels. Scale bars, 10 μm. (D) Quantification of dClover2-positive foci per cell in DC2.4^Tyro3^ (n = 40) and DC2.4^Axl^ (n = 44) shown in (C). Each dot represents a single cell. Horizontal bars indicate the median. (E) Effect of TAM–Gas6 interaction inhibitor RU-302 on the association of CD63-dClover2-sEVs with parental and TAM receptor-expressing LgBiT-DC2.4 cells. Cell-associated sEVs were assessed by flow cytometric analysis of dClover2 median fluorescence intensity (MFI). Data are presented as mean ± SD (n = 4). In panels A, C, and D, DC2.4^Tyro3^, DC2.4^Axl^, and DC2.4^Mertk^ cells are denoted as Tyro3, Axl, and Mertk, respectively. In panels B and E, LgBiT-DC2.4^Tyro3^, LgBiT-DC2.4^Axl^, and LgBiT-DC2.4^Mertk^ cells are denoted as Tyro3, Axl, and Mertk, respectively.

We showed that sEVs bind to the extracellular domains of TAM receptors and that Gas6 or Pros1 differentially enhances their interactions with individual TAM-ECDs. Next, we examined whether TAM receptors could mediate the binding and uptake of sEVs using DC2.4 cells as a dendritic cell model. Flow cytometry analysis did not detect expression of endogenous TAM receptors on the surface of DC2.4 cells under our culture conditions (S1A Fig). Based on this result, we established DC2.4 cells stably expressing Tyro3, Axl, or Mertk (designated DC2.4^Tyro3^, DC2.4^Axl^, and DC2.4^Mertk^, respectively). We confirmed the expression of exogenous TAM receptors in these cell lines by flow cytometry using an anti-HA antibody (S1B Fig). Exogenous Tyro3 in DC2.4^Tyro3^ and exogenous Mertk in DC2.4^Mertk^ were expressed at comparable levels. The expression level of exogenous Axl in DC2.4^Axl^ was higher than those of exogenous Tyro3 and Mertk in DC2.4^Tyro3^ and DC2.4^Mertk^, respectively. We used these engineered cell lines as recipient cells. In the pull-down assay, individual TAM-ECDs showed detectable binding to CD63-NLuc-sEVs even in the absence of exogenous Gas6 or Pros1 supplementation (Fig 1F). We examined sEV association using CD63-NLuc-sEVs without exogenous Gas6 or Pros1 supplementation and quantified cell-associated sEVs based on NLuc activity (Fig 2A). We observed a time-dependent increase in NLuc activity in all recipient cells. NLuc activity in both DC2.4^Axl^ and DC2.4^Mertk^ was approximately 2-fold higher than that in DC2.4 cells. NLuc activity in DC2.4^Tyro3^ was approximately 2-fold higher than that in either DC2.4^Axl^ or DC2.4^Mertk^. DC2.4^Tyro3^ exhibited the highest NLuc activity among the TAM receptor-expressing cell lines. These results indicate that expression of TAM receptors in recipient cells enhances the binding and/or uptake of sEVs. Tyro3 expression had the most pronounced effect under the tested conditions.

To evaluate whether the recipient cells internalize sEVs, the NanoBiT system was employed [24]. This system enabled the detection of CD63-HiBiT-sEVs internalized into LgBiT-expressing recipient cells, as evidenced by NLuc activity resulting from the formation of HiBiT–LgBiT hybrids. Western blot analysis also detected Gas6 and Pros1 in CD63-HiBiT-sEV fractions, together with the sEV-associated proteins CD63 and Alix (Fig 1D). We therefore used CD63-HiBiT-sEVs without exogenous Gas6 or Pros1 supplementation in the following uptake assays. We introduced each TAM receptor into LgBiT-expressing DC2.4 cells (LgBiT-DC2.4) and established stable cell lines co-expressing LgBiT and each TAM receptor (LgBiT-DC2.4^Tyro3^, LgBiT-DC2.4^Axl^, and LgBiT-DC2.4^Mertk^). We assessed the expression levels of individual TAM receptors and LgBiT by flow cytometry. The expression levels of individual TAM receptors and LgBiT were comparable across the cell lines (S1C Fig). We measured NLuc activity in recipient cells over time, which did not increase in LgBiT-DC2.4, LgBiT-DC2.4^Axl^, and LgBiT-DC2.4^Mertk^. In contrast, LgBiT-DC2.4^Tyro3^ showed a time-dependent increase in NLuc activity (Fig 2B). To further confirm sEV internalization, we used CD63-dClover2-sEVs as green fluorescently labeled sEVs to visualize internalized sEVs by fluorescence microscopy. Internalized sEVs were quantified by counting dClover2 foci overlapping with the differential interference contrast (DIC) images of recipient cells (Fig 2C, D). The number of overlapping dClover2 foci in DC2.4^Tyro3^ was 1.6-fold higher than that in DC2.4^Axl^. Although the difference was modest, it was statistically significant. These results show that stable expression of Tyro3 enhances the internalization of sEVs in DC2.4 cells.

To examine the contribution of the Gas6 ligand to TAM receptor-mediated sEV internalization, we used RU-302 as an inhibitor of Gas6-induced TAM receptor activation [25]. Cell-associated CD63-dClover2-sEVs were quantified based on the dClover2 MFI in recipient cells, as measured by flow cytometry (Fig 2E). In LgBiT-DC2.4 cells, the dClover2 MFI was not significantly increased after the addition of CD63-dClover2-sEVs. LgBiT-DC2.4^Tyro3^, LgBiT-DC2.4^Axl^, and LgBiT-DC2.4^Mertk^ showed significant increases in dClover2 MFI after addition of CD63-dClover2-sEVs. DC2.4^Tyro3^ exhibited the highest dClover2 MFI among the TAM-expressing cell lines. In the presence of RU-302, dClover2 MFI was significantly reduced in LgBiT-DC2.4^Tyro3^. LgBiT-DC2.4^Axl^, and LgBiT-DC2.4^Mertk^ showed only limited reductions in dClover2 MFI. These results are consistent with the idea that the Tyro3–Gas6 axis contributes to the association of sEVs with Tyro3-expressing DC2.4 cells. To further examine the effect of RU-302 on sEV uptake, we performed an independent CD63-HiBiT-sEV uptake assay using LgBiT-expressing DC2.4 cell lines (S2 Fig). Consistent with the CD63-dClover2-sEV assay, RU-302 significantly reduced HiBiT–LgBiT luminescence in LgBiT-DC2.4^Tyro3^ cells, whereas only limited or no significant reductions were observed in LgBiT-DC2.4^Axl^ and LgBiT-DC2.4^Mertk^ cells. These findings further support the idea that the Gas6–Tyro3 axis contributes to sEV uptake in Tyro3-expressing DC2.4 cells.

### The IG2c domain is crucial for the association of sEVs with Tyro3

**Fig 3:**
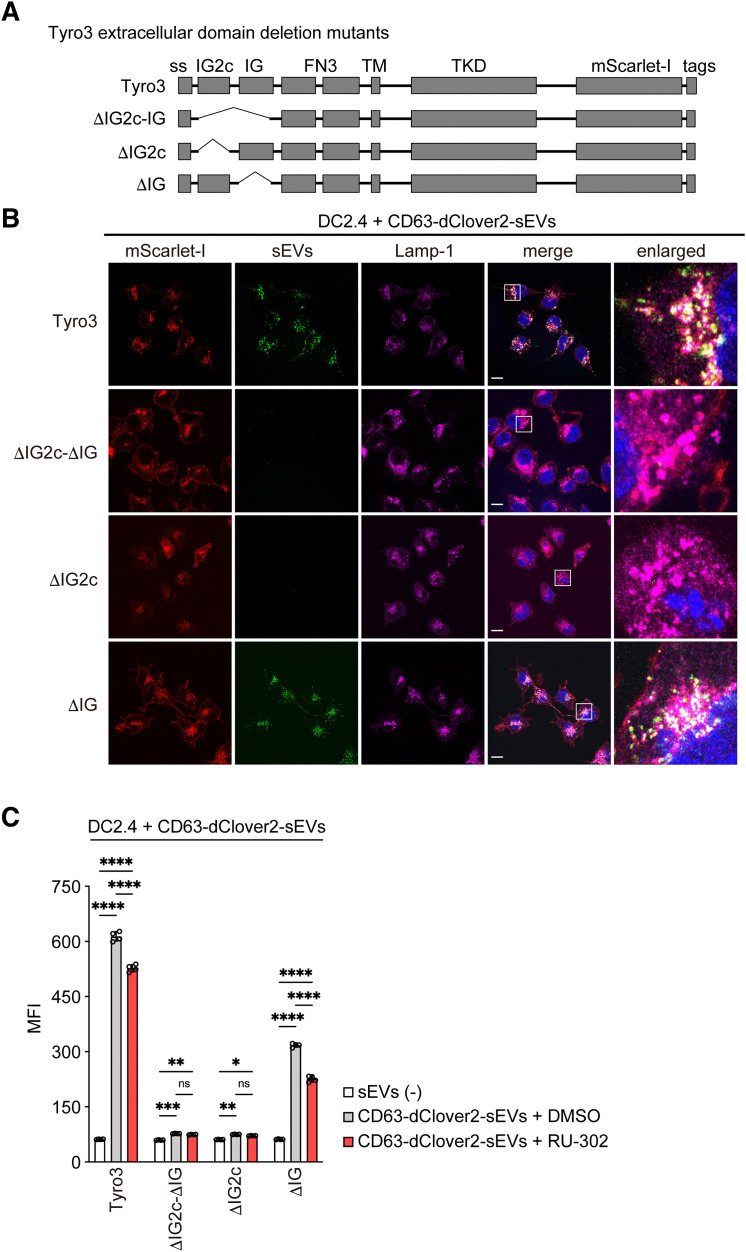
The IG2c domain of Tyro3 contributes to Tyro3-mediated association of sEVs with DC2.4 cells. (A) Schematic diagram of Tyro3 extracellular domain (ECD) deletion mutants. Wavy lines indicate deleted regions: ∆IG2c-IG (aa 45–212), ∆IG2c (aa 45–114), and ∆IG (aa 135–212). Functional domains of mouse Tyro3 were predicted using the Simple Modular Architecture Research Tool (SMART). ss, signal sequence; IG2c, immunoglobulin C2-type domain; IG, immunoglobulin domain; FN3, fibronectin type III domain; TM, transmembrane region; TKD, tyrosine kinase catalytic domain; tags, 3 × HA tag and 6 × His tag. (B) Uptake of CD63-dClover2-sEVs by LgBiT-DC2.4 cells expressing the indicated mScarlet-I-tagged Tyro3 constructs. Tyro3 constructs are shown in red, CD63-dClover2-sEVs in green, Lamp-1 in magenta, and nuclei in blue. Enlarged views of the boxed regions in the merged images are shown on the right. Scale bars, 10 μm. (C) Effect of RU-302 on CD63-dClover2-sEV association with LgBiT-DC2.4 cells expressing the indicated Tyro3 constructs. Cell-associated CD63-dClover2-sEVs were assessed by flow cytometric analysis of dClover2 MFI. Data are presented as mean ± SD (n = 4). In panel A, Tyro3-mScarlet-I, Tyro3-∆IG2c-IG-mScarlet-I, Tyro3-∆IG2c-mScarlet-I, and Tyro3-∆IG-mScarlet-I are denoted as Tyro3, ∆IG2c-IG, ∆IG2c, and ∆IG, respectively. In panels B and C, LgBiT-DC2.4^Tyro3-mScarlet-I^, LgBiT-DC2.4^Tyro3-∆IG2c-IG-mScarlet-I^, LgBiT-DC2.4^Tyro3-∆IG2c-mScarlet-I^, and LgBiT-DC2.4^Tyro3-∆IG-mScarlet-I^ cells are denoted as Tyro3, ∆IG2c-IG, ∆IG2c, and ∆IG, respectively.

To identify which region of the Tyro3 ectodomain is required for the association and uptake of sEVs, we focused on the N-terminal Ig-like domains of Tyro3. Previous structural analysis showed that the N-terminal D1D2 fragment of Tyro3 binds to the Gas6 Laminin-G (LG) like domain [26]. We established LgBiT-DC2.4 cells expressing full-length Tyro3 or Tyro3 mutants lacking Ig-like domains corresponding to the D1D2 region, each fused to mScarlet-I at the C-terminus (LgBiT-DC2.4^Tyro3-mScarlet-I^, LgBiT-DC2.4^Tyro3-∆IG2c-IG-mScarlet-I^, LgBiT-DC2.4^Tyro3-∆IG2c-mScarlet-I^, and LgBiT-DC2.4^Tyro3-∆IG-mScarlet-I^) (Fig 3A). We confirmed that the expression levels of the Tyro3 mutants were comparable to that of full-length Tyro3 across the established cell lines (S1D Fig). LgBiT-DC2.4^Tyro3-mScarlet-I^, LgBiT-DC2.4^Tyro3-∆IG2c-IG-mScarlet-I^, LgBiT-DC2.4^Tyro3-∆IG2c-mScarlet-I^, and LgBiT-DC2.4^Tyro3-∆IG-mScarlet-I^ cells were incubated with CD63-dClover2-sEVs. To examine whether dClover2 signals derived from CD63-dClover2-sEVs localize near the endolysosome, the cells were stained with Lamp-1 (Fig 3B). Under the same conditions, clear dClover2 signals were not detectable in parental DC2.4 cells, and no obvious overlap with Lamp-1-positive compartments was observed (S3 Fig). CD63-dClover2-sEV-derived signals were mainly observed in LgBiT-DC2.4^Tyro3-mScarlet-I^ and LgBiT-DC2.4^Tyro3-∆IG-mScarlet-I^ and partially overlapped with Lamp-1-positive compartments. In contrast, clear dClover2 signals or overlaps with Lamp-1-positive compartments were not detectable in LgBiT-DC2.4^Tyro3-∆IG2c-IG-mScarlet-I^ and LgBiT-DC2.4^Tyro3-∆IG2c-mScarlet-I^ cells. These results indicate that the IG2c domain of Tyro3 contributes to the Tyro3-mediated association of sEVs with DC2.4 cells.

Next, we investigated the effect of RU-302 on sEV association mediated by Tyro3 or its ectodomain deletion mutants. We added CD63-dClover2-sEVs to the recipient cells and quantified cell-associated CD63-dClover2-sEVs based on the dClover2 MFI by flow cytometry (Fig 3C). After the addition of CD63-dClover2-sEVs, LgBiT-DC2.4^Tyro3-mScarlet-I^ and LgBiT-DC2.4^Tyro3-∆IG-mScarlet-I^ showed marked increases in dClover2 MFI, which were reduced by RU-302 treatment. Regardless of RU-302 treatment, LgBiT-DC2.4^Tyro3-∆IG2c-IG-mScarlet-I^ and LgBiT-DC2.4^Tyro3-∆IG2c-mScarlet-I^ cells showed only slight increases in dClover2 MFI following the addition of CD63-dClover2-sEVs. These results further support the contribution of the IG2c domain to Tyro3-mediated sEV association in DC2.4 cells.

### sEV-delivered antigens were cross-presented and activated CTLs

**Fig 4:**
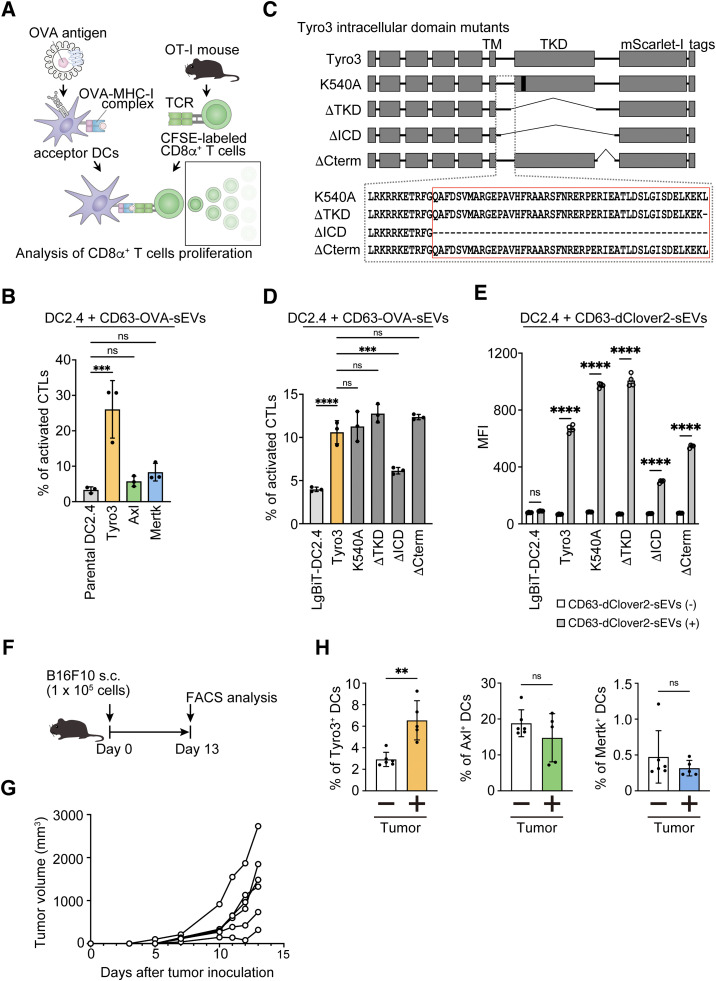
DC2.4 cells that internalized sEVs via Tyro3 cross-present sEV-delivered antigens to CTLs. (A) Schematic diagram of the *in vitro* CTL proliferation assay. DC2.4 cell lines were incubated with CD63-OVA-sEVs and then co-cultured with CFSE-labeled OT-I CD8α ⁺ T cells isolated from OT-I mouse spleens. CTL proliferation was evaluated by CFSE dilution. (B) CTL proliferation induced by parental and TAM receptor-expressing DC2.4 cells after incubation with CD63-OVA-sEVs. Data are presented as mean ± SD (n = 3). (C) Schematic diagram of Tyro3 intracellular domain (ICD) mutants. The black rectangle in K540A indicates the point mutation within the TKD. Wavy lines indicate deleted regions: ∆TKD (aa 508–776), ∆ICD (aa 452–880), and ∆Cterm (aa 782–880). Amino acid sequences immediately downstream of the TM region are shown at the bottom. The sequence corresponding to the Tyro3 antigen presentation-related domain (TAPD) is highlighted with a red rectangle. (D) CTL proliferation induced by parental LgBiT-DC2.4 cells and LgBiT-DC2.4 cells expressing the indicated mScarlet-I-tagged Tyro3 constructs following incubation with CD63-OVA-sEVs. Data are presented as mean ± SD (n = 3). (E) Association of CD63-dClover2-sEVs with parental LgBiT-DC2.4 cells and LgBiT-DC2.4 cells expressing the indicated mScarlet-I-tagged Tyro3 constructs. Cell-associated CD63-dClover2-sEVs were assessed by flow cytometric analysis of dClover2 MFI. Data are presented as mean ± SD (n = 4). (F) Female C57BL/6 mice received a subcutaneous injection of 1 × 105 cells of B16F10 on day 0, and splenocytes from non-tumor-bearing and tumor-bearing mice were analyzed by FACS on day 13. (G) Growth curves of B16F10 tumors. Each line represents an individual mouse (n = 6). (H) Frequencies of Tyro3 ⁺ , Axl ⁺ , and Mertk⁺ cells among splenic DCs (F4/80 3 CD11c ⁺ MHC-II⁺) isolated from non-tumor-bearing and B16F10 tumor-bearing mice on day 13. Data are presented as mean ± SD (n = 6). In panel B, DC2.4^Tyro3^, DC2.4^Axl^, and DC2.4^Mertk^ cells are denoted as Tyro3, Axl, and Mertk, respectively.

The findings described above showed that Tyro3-expressing DC2.4 cells internalized sEVs. Next, we used DC2.4 cells expressing each TAM receptor to examine whether DCs could present sEV-delivered antigens to CTLs. To this end, we used ovalbumin (OVA)-containing sEVs, termed CD63-OVA-sEVs, and CD8α^+^ T cells isolated from OT-I transgenic mice. OVA is a widely used model antigen for analyzing antigen-specific T cell responses. OT-I CD8α^+^ T cells express a transgenic TCR that recognizes the OVA-derived SIINFEKL epitope presented by H-2K^b^. DC2.4, DC2.4^Tyro3^, DC2.4^Axl^, and DC2.4^Mertk^ were pulsed with CD63-OVA-sEVs and subsequently co-cultured with CFSE-labeled CTLs from OT-I mice (Fig 4A). CD8α^+^ T cell proliferation was assessed based on CFSE dilution by flow cytometry (Fig 4B). DC2.4, which lacks TAM receptor expression, activated only about 3% of CTLs. CTL activation induced by DC2.4^Axl^ and DC2.4^Mertk^ was not significantly different from that induced by DC2.4 cells. In contrast, DC2.4^Tyro3^ efficiently induced CTL proliferation, with an approximately 8-fold increase compared with DC2.4 cells. These results show that Tyro3-expressing DC2.4 cells induce stronger activation of OVA antigen-specific CTLs than parental DC2.4 cells.

In panel C, Tyro3-mScarlet-I, Tyro3-K540A-mScarlet-I, Tyro3-ΔTKD-mScarlet-I, Tyro3-ΔICD-mScarlet-I, and Tyro3-ΔCterm-mScarlet-I are denoted as Tyro3, K540A, ∆TKD, ∆ICD, and ∆Cterm, respectively. In panels D and E, LgBiT-DC2.4^Tyro3-mScarlet-I^, LgBiT-DC2.4^Tyro3-K540A-mScarlet-I^, LgBiT-DC2.4^Tyro3-∆TKD-mScarlet-I^, LgBiT-∆C2.4^Tyro3-∆ICD-mScarlet-I^, LgBiT-DC2.4^Tyro3-∆Cterm-mScarlet-I^ cells are denoted as Tyro3, K540A, ∆TKD, ∆ICD, and ∆Cterm, respectively.

### The tyrosine kinase domain of Tyro3 is not required for antigen cross-presentation of sEV-delivered antigens

DC2.4 cells expressing Tyro3 internalize sEVs and activate CTLs via antigen cross-presentation. To identify the intracellular domain (ICD) of Tyro3 involved in this process, DC2.4 cell lines expressing each Tyro3 mutant lacking part of the ICD were established (LgBiT-DC2.4^Tyro3-K540A-mScarlet-I^, LgBiT-DC2.4^Tyro3-∆TKD-mScarlet-I^, LgBiT-DC2.4^Tyro3-∆ICD-mScarlet-I^, and LgBiT-DC2.4^Tyro3-∆Cterm-mScarlet-I^) (Fig 4C). Tyro3-K540A has a point mutation in the ATP-binding site of the tyrosine kinase domain [27]. The expression of these mutants was confirmed by flow cytometry (S1E Fig). We found that the expression levels of Tyro3 variants were comparable across the different ICD mutants. We pulsed those cells with CD63-OVA-sEVs and assessed the activation of CTLs (Fig 4D). We found that the activation was similar among LgBiT-DC2.4^Tyro3-mScarlet-I^, LgBiT-DC2.4^Tyro3-K540A-mScarlet-I^, LgBiT-DC2.4^Tyro3-∆TKD-mScarlet-I^, and LgBiT-DC2.4^Tyro3-∆Cterm-mScarlet-I^, each showing about a 2-fold increase compared with LgBiT-DC2.4 cells. In contrast, LgBiT-DC2.4^Tyro3-∆ICD-mScarlet-I^ exhibited approximately a 50% reduction in CTL activation compared with LgBiT-DC2.4^Tyro3-mScarlet-I^. We identified a 46-amino-acid region that was absent from Tyro3-ΔICD but was retained in Tyro3-ΔTKD and Tyro3-ΔCterm. These findings suggest that this region is important for antigen cross-presentation. This region was designated the Tyro3 antigen presentation-related domain (TAPD).

To evaluate whether the deletion of the ICD affects sEV association, we performed a CD63-dClover2-sEV association assay using LgBiT-DC2.4^Tyro3-mScarlet-I^, LgBiT-DC2.4^Tyro3-K540A-mScarlet-I^, LgBiT-DC2.4^Tyro3-∆TKD-mScarlet-I^, LgBiT-DC2.4^Tyro3-∆ICD-mScarlet-I^, and LgBiT-DC2.4^Tyro3-∆Cterm-mScarlet-I^ cells (Fig 4E). LgBiT-DC2.4^Tyro3-∆ICD-mScarlet-I^ cells showed a significant increase in dClover2 MFI after the addition of CD63-dClover2-sEVs, although the dClover2 MFI was lower than that observed in cells expressing full-length Tyro3. These results indicate that deletion of the entire intracellular domain reduces the association of CD63-dClover2-sEVs with Tyro3-expressing DC2.4 cells. However, this deletion did not completely abolish sEV association.

DC2.4 cells expressing Tyro3 activated CTLs upon antigen delivery by sEVs through the Tyro3 receptor. Finally, we analyzed the expression levels of TAM receptors in splenic DCs using a B16F10 tumor-bearing mouse model. We subcutaneously injected mice with 1 × 105 B16F10 cells and analyzed splenic DCs 13 days later (Fig 4F, G). We analyzed splenic DCs defined as F4/80 3 CD11c ⁺ MHC-II⁺ cells. These DCs can present antigens. We compared TAM receptor-positive populations within DCs between non-tumor-bearing and tumor-bearing mice (Fig 4H). The frequencies of Axl^+^ or Mertk^+^ DCs were similar regardless of tumor presence. However, the proportion of Tyro3 ⁺ DCs was about 2- to 3-fold higher in tumor-bearing mice than in non-tumor-bearing mice. Under tumor-bearing conditions, the proportion of Tyro3-expressing DCs increased in the spleen.

## Discussion

To our knowledge, we demonstrated for the first time that Tyro3 contributes to sEV association/internalization and antigen cross-presentation in DCs, based on experiments using a dendritic cell line exogenously expressing Tyro3. TAM receptors are important regulators of immune homeostasis in dendritic cells and other antigen-presenting cells [17]. However, we did not detect endogenous surface expression of Tyro3, Axl, or Mertk in parental DC2.4 cells by flow cytometry under our assay conditions. Previous studies have shown that the expression patterns of individual TAM receptors vary among bone marrow-derived DCs (BMDCs) and DC populations. Axl and Mertk, but not Tyro3, were detected in Flt3L-induced BMDCs and splenic CD11c^+^ DCs, whereas Tyro3 was detected in PD-L2^+^ and PD-L2^−^ DCs from GM-CSF-induced BMDC cultures [28,29]. These findings suggest that the expression patterns of individual TAM receptors appear to be influenced by differentiation state, activation status, cellular subset, inflammatory context, and detection method. Thus, the lack of detectable endogenous TAM receptors in DC2.4 cells may reflect low basal expression below the detection limit and/or characteristics of DC2.4 cells as an immortalized dendritic cell line. Therefore, we used DC2.4 cells individually expressing exogenous TAM receptors to examine the involvement of each receptor in sEV association and internalization.

We assessed whether TAM receptors contribute to the internalization of sEVs. We found that Tyro3 enhances sEV uptake in DC2.4 via the ligands Gas6 or Pros1. We also showed that the IG2c domain of Tyro3 is required for efficient sEV association and internalization, as DC2.4 cells expressing intact Tyro3 internalized sEVs, whereas those expressing the Tyro3-∆IG2c mutant showed only minimal sEV-associated signals. The inability of the Tyro3-∆IG2c mutant to associate with sEVs likely results from impaired interaction with Gas6 or Pros1, as the Immunoglobulin-like (Ig-like) D1D2 domain of Tyro3 is responsible for Gas6 binding [26]. Consistent with this interpretation, RU-302 partially reduced the association of sEVs in cells expressing intact Tyro3 or Tyro3-ΔIG mutant. RU-302 has been reported to inhibit Gas6-induced activation of TAM receptors by targeting the interface between the TAM Ig-like domain and the Gas6-LG domain [25]. It should be noted that the inhibitory effect of RU-302 was partial in cells expressing intact Tyro3 or Tyro3-ΔIG. This partial inhibition may be explained by the contribution of Pros1-dependent interactions, incomplete blockade of vesicle-associated Gas6 under our experimental conditions, or by other RU-302-insensitive mechanisms may also contribute to sEV association.

Here, we show weak binding between Tyro3 and PG under our assay conditions, suggesting that Tyro3 may recognize PS and PG on sEVs. While prior studies detected Gas6 and Pros1 binding to PS by phospholipid overlay assay, PG binding was not observed, likely due to the use of a lower amount (74.5 ng or 100 ng) [30,31]. In contrast, this study used a larger amount of PG (800 ng), enabling the detection of Tyro3-ECD binding to PG via Gas6 or Pros1.

PS, which is abundant in the plasma membrane, is also rich in sEV membranes. In contrast, PG is less abundant in the plasma membrane and typically localizes to the inner mitochondrial membrane [22,32,33]. Cardiolipin (CL), a mitochondrial-specific phospholipid formed from PG dimers, has been detected in mitochondria-derived extracellular vesicles (mitovesicles) [34]. CL at approximately 74.5 ng may interact weakly with Gas6. This interpretation is based on a careful inspection of the published data, although it was not explicitly mentioned by the authors [31]. PG and CL are also components of the membranes of Gram-positive bacteria [35,36]. Among these, *Streptococcus pneumoniae* and *Streptococcus agalactiae* are pathogens of bacterial meningitis. Tyro3 is not only expressed in DCs and macrophages, but also shows high expression in the brain (S4 Fig). Thus, the weak interaction of Tyro3 with PG via Gas6 may imply a possible involvement of the Gas6–Tyro3 axis in mitovesicle uptake, suggesting that this system could function as a pattern recognition receptor in the context of infection, including bacterial meningitis. Further investigation is needed to clarify the underlying mechanisms for this speculation.

TAM receptors play roles in immunosuppression and immune evasion in the tumor microenvironment [37]. Axl is upregulated by TLR-induced IFNAR/STAT1 signaling and subsequently induces SOCS1/3 to suppress further TLR signaling in DCs [28]. In tumor-bearing mice, the expression of TAM receptors was upregulated in myeloid-derived suppressive cells (MDSCs) [38]. Indeed, inhibitors targeting TAM receptors are currently under clinical development [39].

In contrast, this study demonstrates that Tyro3 contributes to the cross-presentation of sEV-delivered antigens, which requires the TAPD but not the kinase domain. The TAPD is highly conserved across mammals, indicating its functional importance (S5 Fig). Notably, deletion of the entire intracellular domain of Tyro3 reduced, but did not abolish, the association of sEVs with Tyro3-expressing DC2.4 cells. These findings suggest that the TAPD-containing intracellular region of Tyro3 may contribute to efficient antigen cross-presentation. This region may also affect sEV association and/or intracellular trafficking, although the precise mechanism remains unclear. In a previous study, the Axl/LRP-1/RANBP9 complex in BMDCs was shown to facilitate efferocytosis and antigen cross-presentation [40]. The interaction between Axl and LRP-1 requires RANBP9, and it also associates with human Tyro3-ICD (aa 452–890), which includes the TAPD [41]. Additionally, Tyro3 binds to Fyn through its intracellular domain (aa 451–890), which also encompasses the TAPD, thereby regulating Schwann cell myelination [42]. Fyn has been detected in DC2.4 cells by proteomic analysis of palmitoylated proteins, indicating the expression of this kinase in this cell line [43]. Although our study did not provide direct evidence, these observations raise the possibility that the TAPD of Tyro3 may interact with intracellular molecules, including LRP-1, RANBP9, and Fyn. Moreover, TAM receptors are required for NK cell differentiation and cytotoxicity [44]. MerTK signaling functioned as a late costimulatory signal to CD8 ⁺ T cells [45]. Similarly, Tyro3 may enhance antigen cross-presentation in DC2.4 cells in a context-dependent manner.

Notably, in our study, the frequency of Tyro3 ⁺ splenic DCs (CD11c ⁺ , F4/80 3 , MHC-II⁺) was increased in tumor-bearing mice. In DC2.4 model system, DC2.4^Tyro3^ that internalized sEVs did not produce detectable levels of pro-inflammatory cytokines IL-6 and TNF-α (S6 Fig). Thus, Tyro3 may represent a potential target for EV-based antigen delivery to splenic DCs. Several early-phase (Phase I/II) EV-based clinical trials have demonstrated acceptable safety profiles [46–48]. However, T-cell responses have generally been limited, with effects largely dependent on NK cell activity. Understanding Tyro3-mediated EV uptake by DCs may provide insights into cancer immunotherapy and could help inform future EV-based vaccine strategies.

## Conclusion

This study identifies Tyro3 as a receptor that mediates the uptake of small extracellular vesicles (sEVs) and enables antigen cross-presentation in dendritic cells, using the DC2.4 model system. We demonstrate that Tyro3 recognizes sEVs through the bridging ligands Gas6 or Pros1 and that efficient internalization requires the IG2c domain of its extracellular region. Furthermore, we show that the 46-amino acid intracellular region of Tyro3, designated the Tyro3 antigen presentation-related domain (TAPD), contributes to efficient cross-presentation of sEV-delivered antigens. The increased frequency of Tyro3 ⁺ dendritic cells in tumor-bearing mice further supports a potential role for this pathway *in vivo*. Based on these findings, we propose a model in which Tyro3 promotes sEV uptake and contributes to antigen cross-presentation in dendritic cells (S7 Fig). Together, these findings establish Tyro3 as a candidate target of sEV-mediated antigen delivery and highlight its potential utility in sEV-based cancer immunotherapy.

## Materials and methods

### Cell lines and culture conditions

Expi293F was purchased from Thermo Fisher Scientific and cultured according to the manufacturer’s instructions. Expi293F cells are human 293-derived suspension cells used for transient expression and sEV production. 293T cells were obtained from American Type Culture Collection (ATCC). 293T cells are a human embryonic kidney 293-derived cell line stably expressing the SV40 large T antigen. The 293 lineage has been reported to be of female provenance based on the absence of detectable Y-chromosome-derived sequences [49]. B16F10 cells were obtained from the RIKEN BioResource Research Center (BRC). B16F10 cells are a C57BL/6-derived murine melanoma cell line and have been reported to be of female origin, based on the absence of detectable sequencing reads aligned to the Y chromosome [50]. B16F10 cells were used for the tumor model. 293T and B16F10 cells were cultured in D-MEM. DC2.4 cells, derived from the bone marrow of C57BL/6 mice, were kindly provided by Dr. Atsuko Asao (Tohoku University). DC2.4 cells are an immortalized murine dendritic cell line originally established from C57BL/6 mouse bone marrow cultures by transduction with murine GM-CSF followed by immortalization with myc and raf oncogenes [51]. The sex of the donor mouse used to establish this cell line was not specified in the original report. DC2.4 cells were cultured in RPMI-1640 medium. For sEV addition experiments to the cell cultures, we used media containing EV-depleted fetal bovine serum (FBS) prepared by ultracentrifugation to remove EVs.

### Animal experiments

C57BL/6 mice and OT-I mice were purchased from Japan SLC and Charles River Laboratories, respectively. Seven-week-old female C57BL/6 mice (mean weight, 18 g) were used for subcutaneous inoculation of B16F10 cells. Eight- to ten-week-old female OT-I mice (mean weight, 19–20 g) were used for isolation of CD8α^+^ T cells from spleens. C57BL/6 mice were anesthetized with isoflurane vapor (2% in oxygen; 099–06571, FUJIFILM Wako) using a nose cone for subcutaneous inoculation of B16F10 cells. Tumor sizes were measured using standard calipers, and tumor volume (mm^3^) was calculated using the formula: 0.5 × length × width^2^. Animals were euthanized when the tumor volume reached 2000 mm³. All tumor-bearing mice were humanely euthanized on Day 13 in accordance with predefined humane endpoint criteria, and spleens were collected for FACS analysis. For euthanasia, mice were exposed to an overdose of isoflurane vapor (5% in oxygen) in a closed chamber without prior induction of anesthesia until they stopped breathing. Cervical dislocation was then performed to ensure death. All mice were maintained under specific pathogen-free conditions following guidelines for animal experimentation. All animal experiments were conducted in accordance with the guidelines of the Miyagi Cancer Center Animal Experiment Committee. The study protocol was approved by the committee under approval number AE.25.04, and the study was conducted in compliance with the ARRIVE guidelines 2.0. No human participants, primary human samples, or identifiable human data were used in this study. Established cell lines were used as described above; therefore, informed consent was not applicable.

### Virus transduction

We established DC2.4 cell lines stably expressing fusion proteins by lentiviral transduction. The fusion proteins expressed in DC2.4 cells are summarized below. We generated the following constructs: (i) mouse Tyro3 (NP_062265.2), Axl (NP_001177903.1), and Mertk (NP_032613.1) tagged at their C-termini with 3 × HA and 6 × His; (ii) mouse Tyro3 and its seven mutants fused at their C-termini with mScarlet-I, 3 × HA, and 6 × His; (iii) LgBiT tagged at its C-termini with 3 × FLAG. Lentiviral particles were produced by transfecting 293T cells with plasmids using the polyethylenimine method, as described in our previous methods [52]. Stable cell lines were selected using 2 μg/mL blasticidin or 600 μg/mL G418 (both from FUJIFILM Wako), followed by limiting dilution or single-cell sorting using a SONY cell sorter (MA900).

### Preparation of recombinant proteins

The expression plasmids used in this study for the preparation of recombinant proteins are summarized below (S1 Table). We generated the following constructs: (i) mouse Gas6 and Pros1 tagged at their C-termini with 6 × His; (ii) mouse Tyro3 extracellular domain (ECD) (aa 25–412), mouse Axl-ECD (aa 19–443), and mouse Mertk-ECD (aa 23–497) tagged at their C-termini with Myc and 7 × His. The expression constructs for Tyro3-ECD, Axl-ECD, and Mertk-ECD contained an artificial signal sequence [53]. We used the Expi293F Expression System and the ExpiFectamine 293 Transfection Kit (Thermo Fisher Scientific) to produce recombinant proteins, following the manufacturer’s instructions. For the expression of Gas6 and Pros1, 10 μg/mL vitamin K1 (Nacalai Tesque) was added to the Expi293F culture medium [20]. Recombinant proteins were purified using HisTrap HP and PD-10 desalting columns (GE Healthcare, now Cytiva).

### Preparation of sEVs

To prepare reporter- or antigen-labeled sEVs, we generated the following constructs: (i) mouse CD63 fused at its C-termini with NanoLuc (NLuc) luciferase, 3 × HA, and 6 × His [54]; (ii) mouse CD63 tagged at its C-termini with HiBiT tag; (iii) mouse CD63 fused at their C-termini with dClover2, Myc, and 6 × His; (iv) mouse CD63 fused at their C-termini with ovalbumin (OVA), Myc, and 6 × His. sEVs generated using constructs (i) to (iv) were designated as CD63-NLuc-sEVs, CD63-HiBiT-sEVs, CD63-dClover2-sEVs, and CD63-OVA-sEVs, respectively. These fusion proteins were overexpressed under serum-free conditions using the Expi293F Expression System (Thermo Fisher Scientific) according to the manufacturer's instructions. The culture supernatant was centrifuged at 1,500 rpm for 30 min to remove cells and cell debris, followed by filtration through a 0.22 μm membrane (Thermo Fisher Scientific) to exclude extracellular vesicles exceeding 220 nm in diameter. We monitored the filtered culture supernatant for particle concentration and size distribution using the NTA method with a Malvern NanoSight NS300 (Malvern Panalytical) [55]. sEVs were purified using the differential ultracentrifugation (dUC) method [56]. The supernatant was first centrifuged at 10,000 × g for 30 min at 4 °C to remove cell debris. The resulting supernatant was then ultracentrifuged at 100,000 × g for 70 min at 4 °C using a Beckman Coulter Optima XE-90 ultracentrifuge with a SW32Ti rotor. The pellet containing sEVs was washed once with PBS and subjected to a second ultracentrifugation at 100,000 × g for 70 min. The final pellet was resuspended in PBS. For TEM analysis, sEV samples were stained with platinum blue using a TI-blue staining kit (Nisshin EM) and observed using a JEM-1400 microscope (JEOL), as previously reported [57]. To prepare Gas6- or Pros1-bound sEVs, we opsonized CD63-NLuc-sEVs with 1 mM Gas6 or Pros1 in the presence of 2 mM CaCl_2_ and 1 mM MgCl_2_. We termed Gas6- and Pros1-opsonized sEVs as +Gas6-sEVs and +Pros1-sEVs, respectively. The amounts of all sEVs used in this study were quantified using a BCA Protein Assay Kit (Nacalai Tesque). In addition, we measured NLuc-dependent and HiBiT-dependent luciferase activities in NLuc- or HiBiT-labeled sEVs, respectively.

### Pull-down assay

Anti-Myc tag monoclonal antibody (MBL, clone My3) was bound to Dynabeads Protein G (Thermo Fisher Scientific) in blocking buffer (TBS containing 2 mM CaCl2 and 1% BSA) at room temperature for 20 min. After the supernatant was removed, Tyro3-ECD, Axl-ECD, or Mertk-ECD was added to the antibody-bound beads at 100 nM and incubated at 4 °C for 60 min. After the supernatant was removed again, an equal amount of CD63-NLuc-sEVs, + Gas6-sEVs, or +Pros1-sEVs was added to the TAM-ECD-bound beads and incubated at 4 °C for 60 min. The amount of sEVs was normalized by NLuc luciferase activity, as a previous report demonstrated that NLuc luminescence correlates with exosome particle number [58]. The beads were then washed three times with blocking buffer to remove unbound sEVs. We added 100 μL of 1 × Glo Lysis Buffer (Promega) to the beads and quantified TAM-ECD-bound sEVs by measuring the levels of NLuc-dependent luciferase activity.

### Phospholipid overlay assay

We used the following phospholipids in this study: phosphatidylserines (PS, soy), phosphatidylcholines (PC, soy), phosphatidylglycerols (PG, egg), phosphatidylethanolamines (PE, bovine), and sphingomyelins (SM, egg, all from Cayman Chemical). We serially diluted the phospholipids in a solvent mixture of methanol, chloroform, and ultrapure water (2:1:1, v/v/v). The diluted phospholipids were spotted onto Hybond-C nitrocellulose membranes (Amersham) at 800 ng, 253 ng, 80 ng, and 25 ng. The membranes were air-dried at room temperature for 60 min and then blocked with blocking buffer (TBS containing 2 mM CaCl2 and 1% BSA) at room temperature for 60 min. The membranes were washed three times with wash buffer (TBS containing 2 mM CaCl2 and 0.02% Tween-20) and then incubated with 100 nM Gas6 or 100 nM Pros1 at room temperature for 60 min. The membranes were washed three times with wash buffer and then incubated overnight at 4 °C with 100 nM Tyro3-ECD, Axl-ECD, or Mertk-ECD in reaction buffer (TBS containing 2 mM CaCl2). Gas6 and Pros1 directly bound to phospholipids were detected using an HRP-conjugated anti-His tag antibody (MBL, OGHis; 1:5000). TAM-ECDs indirectly bound to phospholipids via Gas6 or Pros1 were detected using an HRP-conjugated anti-Myc tag monoclonal antibody (MBL, My3; 1:10,000 dilution). Chemiluminescence was detected using SuperSignal West Pico PLUS Chemiluminescent Substrate (Thermo Fisher Scientific) and an ImageQuant LAS 4000 mini imaging system (Cytiva).

### Flow cytometry

DC2.4 cell lines and splenocytes from C57BL/6 mice were pre-incubated with anti-CD16/32 antibody to block Fc receptors. Subsequently, dead cells were stained with 7-AAD (Sigma), Zombie NIR Viability Kit (BioLegend), or Zombie Aqua Viability Kit (BioLegend). The cells were washed three times with FACS buffer (PBS containing 2% FBS), followed by extracellular staining. For intracellular staining, the cells were washed with FACS buffer after dead cell staining and fixed in the dark with 4% PFA for 30 min at 4 °C. The fixed cells were permeabilized by washing three times with Intracellular Staining Permeabilization Wash Buffer (BioLegend) and intracellularly stained in permeabilization buffer. See antibodies used for flow cytometry in S2 Table. We used a SONY Cell Analyzer SA3800 and analyzed the data using FlowJo software (version 10.10.0; BD Biosciences).

### sEV binding and internalization assay on cultured cells

Recipient cells were seeded at 5 × 10^4^ cells per well in U-bottom 96-well plates and cultured overnight in the presence of 500 ng/mL LPS. CD63-NLuc-sEVs or CD63-HiBiT-sEVs were then added and incubated at 37 °C for the indicated time points. The amount of sEVs was normalized by luminescence. The cells were washed three times with FACS buffer to remove unbound sEVs and lysed in 1 × Glo Lysis Buffer (Promega). We quantified the sEVs interacting with the cells by measuring the levels of NLuc- or HiBiT-dependent luciferase activity using a Synergy H1 microplate reader (BioTek) or a TriStar3 microplate reader (Berthold Technologies). For assays using CD63-dClover2-sEVs, recipient cells were seeded at 5 × 10^4^ cells per well in U-bottom 96-well plates and cultured overnight without LPS. CD63-dClover2-sEVs were pre-incubated overnight at 4 °C with either DMSO or 20 μM RU-302. CD63-dClover2-sEVs were added at a concentration of 20 μg/mL and incubated at 37 °C for 3 hours in the presence of DMSO or 20 μM RU-302. The cells were washed three times with FACS buffer to remove unbound sEVs and analyzed using the SA3800 cell analyzer.

### Western blot

CD63-NLuc-sEVs and CD63-HiBiT-sEVs were lysed in 1 × Glo Lysis Buffer (Promega). We prepared lysates of organs from C57BL/6 mice by homogenization and sonication in Lysis buffer (1% Triton X-100, 150 mM NaCl, 20 mM Tris, 2.5 mM sodium pyrophosphate, 1 mM EDTA, 1 mM EGTA, 1 mM β-glycerol phosphate). These lysates were separated by SDS-PAGE and transferred onto a PVDF membrane (Millipore). We blocked the membrane with blocking buffer (TBS containing 5% skim milk and 0.1% Tween-20) and incubated it with a primary antibody in wash buffer (TBS containing 0.1% Tween-20), followed by washing with wash buffer and incubation with a secondary antibody. The antibodies used for WB are listed in S2 Table. Chemiluminescence was detected using SuperSignal West Pico PLUS Chemiluminescent Substrate and an ImageQuant LAS 4000 mini imaging system or FUSION SOLO S imaging system (Vilber).

### Immunofluorescence microscopy

Recipient cells were seeded at a density of 3 × 10^4^ cells on glass coverslips and cultured overnight. CD63-dClover2-sEVs were added at 20 μg/mL and incubated at 37 °C for 2 hours. Cells were fixed with ice-cold methanol for 3 min or 4% PFA for 10 min at room temperature. Following PFA treatment, we permeabilized cells with 0.1% Triton X-100 in PBS for 3 min at room temperature. To block nonspecific binding, DC2.4 cells were treated with 10% FBS in PBS at 25 °C for 60 min. We incubated cells with the indicated primary antibodies (1 μg/mL) at 4 °C overnight. After washing, the samples were incubated with secondary antibodies (1 μg/mL) at 37 °C for 1 hour. We stained nuclei with DAPI. The antibodies used for immunofluorescence are listed in S2 Table. We captured fluorescence images using an FV3000 confocal microscope (EVIDENT) or an LSM880 Airyscan confocal microscope (Carl Zeiss).

### *In vitro* CTL activation assay

CD8α^+^ T cells were isolated from the spleens of OT-I transgenic mice using a CD8α^+^ T Cell Isolation Kit for mice (Miltenyi). We stained the CD8α^+^ T cells with 2 mM Carboxyfluorescein Succinimidyl Ester (CFSE) (Thermo Fisher Scientific). DC2.4 cell lines were stimulated with 500 ng/mL LPS and 10 μg/mL CD63-OVA-sEVs at 37 °C overnight. The next day, we treated them with 10 μg/mL mitomycin C for 2 hours at 37 °C and then washed them with RPMI-1640 medium. Subsequently, we seeded 1 × 10^4^ recipient cells per well in a U-bottom 96-well plate and added CFSE-labeled CD8α^+^ T cells at 1 × 10^5^ cells per well. After 3 days of co-culture, T cell proliferation was assessed based on the dilution of CFSE fluorescence using a SONY Cell Analyzer SA3800.

### Statistical analysis

Statistical analysis was performed using GraphPad Prism 10 version 10.2.2. Comparisons between two groups were conducted using Student's *t-*test. For comparisons among three or more groups, one-way or two-way ANOVA with Sidak’s multiple comparisons test was used. Statistical significance was defined as *p < 0.05, **p < 0.01, ***p < 0.001, or ****p < 0.0001.

## Supporting information

S1 Fig Expression patterns of TAM receptors in DC2.4 cell lines. The expression of TAM receptors, Tyro3 mutants, and LgBiT was analyzed by flow cytometry. (A) Endogenous cell-surface expression of Tyro3, Axl, and Mertk in parental DC2.4 cells. Cells were stained with antibodies against each TAM receptor. Red lines indicate specific antibody staining. (B) Expression of C-terminally HA-tagged Tyro3, Axl, and Mertk in DC2.4 cell lines, detected by intracellular anti-HA staining. Red lines indicate each TAM receptor-expressing cell line, and blue lines indicate parental DC2.4 cells. (C) Expression of HA-tagged TAM receptors and FLAG-tagged LgBiT in LgBiT-DC2.4-based cell lines. Cells were intracellularly stained with anti-HA antibody to detect HA-tagged TAM receptors (upper panels) or with anti-FLAG antibody to detect FLAG-tagged LgBiT (lower panels). Red lines indicate each LgBiT-DC2.4 cell line expressing the individual TAM receptor, blue lines indicate LgBiT-DC2.4 cells, and black lines indicate parental DC2.4 cells. (D) Expression of mScarlet-I-tagged Tyro3 and Tyro3 extracellular domain deletion mutants in LgBiT-DC2.4 cell lines, assessed by mScarlet-I fluorescence. Red lines indicate cells expressing the indicated Tyro3 construct, blue lines indicate LgBiT-DC2.4 cells, and gray filled histograms indicate negative controls. (E) Expression of mScarlet-I-tagged Tyro3 intracellular domain mutants in LgBiT-DC2.4 cell lines, assessed by mScarlet-I fluorescence. Red lines indicate cells expressing the indicated Tyro3 construct, and blue lines indicate LgBiT-DC2.4 cells. In panels A, B, and C, gray filled histograms indicate isotype controls. In panels B and C, Tyro3, Axl, and Mertk indicate the corresponding TAM receptor-expressing DC2.4 or LgBiT-DC2.4 cells. In D and E, construct labels indicate the corresponding mScarlet-I-tagged Tyro3 constructs: Tyro3, ∆IG2c-IG, ∆IG2c, ∆IG, K540A, ∆TKD, ∆ICD, and ∆Cterm correspond to Tyro3-mScarlet-I, Tyro3-∆IG2c-IG-mScarlet-I, Tyro3-∆IG2c-mScarlet-I, Tyro3-∆IG-mScarlet-I, Tyro3-K540A-mScarlet-I, Tyro3-∆TKD-mScarlet-I, Tyro3-∆ICD-mScarlet-I, and Tyro3-∆Cterm-mScarlet-I, respectively.
(TIF)

S2 Fig Effect of RU-302 on CD63-HiBiT-sEV uptake in DC2.4 cells. DC2.4, LgBiT-DC2.4, LgBiT-DC2.4^Tyro3^, LgBiT-DC2.4^Axl^, and LgBiT-DC2.4^Mertk^ cells were used as recipient cells. Recipient cells and CD63-HiBiT-sEVs were pretreated with DMSO or RU-302 (20 μM) overnight. These cells were then incubated with the sEVs at 37 °C for 6 h in the continued presence of DMSO or RU-302 (20 μM). CD63-HiBiT-sEV uptake was evaluated by measuring luminescence generated by HiBiT–LgBiT complementation. Data are presented as mean ± SD (n = 4). In the figure, Tyro3, Axl, and Mertk indicate DC2.4 cells stably expressing the corresponding TAM receptor.
(TIF)

S3 Fig Confocal imaging of parental DC2.4 cells incubated with CD63-dClover2-sEVs. Uptake of CD63-dClover2-sEVs by parental DC2.4 cells. The mScarlet-I fluorescence channel was acquired as a negative-control channel and is shown in red. CD63-dClover2-sEVs are shown in green. Lamp-1 is shown in magenta. Nuclei were counterstained with DAPI (blue). Enlarged views of the boxed regions in the merged images are shown on the right. Scale bars, 10 μm.
(TIF)

S4 Fig Expression pattern of endogenous Tyro3 in organs of C57BL/6 mice. Endogenous Tyro3 expression in bronchoalveolar lavage fluid, peritoneal macrophages, spleen, thymus, inguinal lymph nodes, liver, kidney, and heart was analyzed by Western blotting. We used GAPDH as a loading control.
(TIF)

S5 Fig Alignment of mammalian Tyro3 amino acid sequences. Full-length amino acid sequences of mammalian Tyro3 were aligned using Clustal Omega. The Tyro3 antigen presentation-related domain (TAPD) region in mouse Tyro3 is highlighted with a red rectangle. Functional domains of human Tyro3 were predicted using the SMART program. The amino acid sequences corresponding to the predicted functional domains are highlighted with black rectangles. The accession numbers of the Tyro3 sequences used for alignment are as follows: Human (NP_006284.2), Monkey (XP_014997475), Cow (DAA25396), Dog (XP_038297974), Rat (NP_058788), and Mouse (NP_062265.2). ss, signal sequence; IG2c, Immunoglobulin C-2 Type domain; IG, Immunoglobulin domain; FN3, Fibronectin type 3 domain; TM, transmembrane region; TKD, tyrosine kinase catalytic domain.
(TIF)

S6 Fig Pro-inflammatory cytokine production by sEV-stimulated DC2.4 cells. The amounts of mouse IL-6 (A) and mouse TNF-α (B) secreted into the culture supernatant by DC2.4 cells are shown. DC2.4 and DC2.4^Tyro3^ were seeded at 2.9 × 105 cells per well in 24-well plates and stimulated overnight with 10 μg/mL CD63-NLuc-sEVs or 500 ng/mL LPS. Culture supernatants were collected, and pro-inflammatory cytokine levels were quantified by ELISA using ELISA MAX Deluxe Set Mouse IL-6 (BioLegend) and ELISA MAX Deluxe Set Mouse TNF-α (BioLegend), according to the manufacturer's instructions.
(TIF)

S7 Fig Proposed model of cross-presentation of sEV-delivered antigens by Tyro3-expressing dendritic cells. The schematic illustrates a proposed pathway by which Tyro3-expressing dendritic cells cross-present sEV-delivered antigens. The numbered labels indicate the following proposed steps. (1) In this study, CD63-OVA-sEVs were used as model antigen-carrying sEVs. Antigen-carrying sEVs interact with Tyro3 through Gas6 and/or Pros1 and are internalized by dendritic cells. (2) Internalized sEVs localize near Lamp-1-positive endolysosomal compartments. (3) The antigens delivered by sEVs are processed. (4) Processed antigens are cross-presented via MHC class I molecules to CD8α ⁺ T cells. (5) The TAPD, a 46-amino-acid region immediately downstream of the transmembrane domain, plays an important role in this pathway. These findings raise the possibility that unknown factor(s) may cooperate with Tyro3 to promote efficient sEV association and/or antigen cross-presentation.
(TIF)

S8 Fig Original western blot data for Figure 1D. Original western blot images corresponding to Fig 1D are shown. Boxes indicate the regions cropped and presented in Fig 1D.
(TIF)

S9 Fig Original data on phospholipid overlay assay in this study. Original blot data obtained by long exposure corresponding to Fig 1I are shown. Boxes indicate the regions cropped and presented in Fig 1I.
(TIF)

S10 Fig Original western blot data for S4 Fig. Original western blot images corresponding to S4 Fig are shown. Boxed regions indicate the areas cropped and presented in S4 Fig.
(TIF)

S1 Table Expression plasmids used for preparation of recombinant proteins in this study. (DOCX)

S2 Table Antibodies used in this study. (DOCX)

S1 Data Raw numerical data underlying the figures presented in this study. Each sheet corresponds to the figure indicated by the sheet name.
(XLSX)

## Abbreviations

TAM: Tyro3, Axl, and Mertk
DCs: Dendritic cells
PS: Phosphatidylserine
PC: Phosphatidylcholine
PG: Phosphatidylglycerol
PE: Phosphatidylethanolamine
SM: Sphingomyelin
NLuc: NanoLuc luciferase
EVs: Extracellular vesicles
sEVs: Small extracellular vesicles
ECD: Extracellular domain
ICD: Intracellular domain
CTLs: Cytotoxic T lymphocytes
APCs: Antigen-presenting cells
DDS: Drug delivery system

## References

[1] Kalluri R, LeBleu VS. The biology, function, and biomedical applications of exosomes. Science. 2020;367(6478):eaau6977. doi: 10.1126/science.aau6977 32029601 PMC7717626

[2] Welsh JA, Goberdhan DCI, O’Driscoll L, Buzas EI, Blenkiron C, Bussolati B, et al. Minimal information for studies of extracellular vesicles (MISEV2023): From basic to advanced approaches. J Extracell Vesicles. 2024;13(2):e12404. doi: 10.1002/jev2.12404 38326288 PMC10850029

[3] Barnwal A, Gaur V, Sengupta A, Tyagi W, Das S, Bhattacharyya J. Tumor Antigen-Primed Dendritic Cell-Derived Exosome Synergizes with Colony Stimulating Factor-1 Receptor Inhibitor by Modulating the Tumor Microenvironment and Systemic Immunity. ACS Biomater Sci Eng. 2023;9(11):6409–24. doi: 10.1021/acsbiomaterials.3c00469 37870457

[4] Kanuma T, Yamamoto T, Kobiyama K, Moriishi E, Masuta Y, Kusakabe T, et al. CD63-Mediated Antigen Delivery into Extracellular Vesicles via DNA Vaccination Results in Robust CD8+ T Cell Responses. J Immunol. 2017;198(12):4707–15. doi: 10.4049/jimmunol.1600731 28507029

[5] Xia J, Miao Y, Wang X, Huang X, Dai J. Recent progress of dendritic cell-derived exosomes (Dex) as an anti-cancer nanovaccine. Biomed Pharmacother. 2022;152:113250. doi: 10.1016/j.biopha.2022.113250 35700679

[6] Safarzadeh M, Saadat N, Abbasi-Molaei S, Rastegari-Pouyani M. Extracellular vesicles as missiles for enhanced anti-tumor efficacy of oncolytic viruses: from disseminating oncolysis and anti-tumor immunity to targeted delivery. Cell Commun Signal. 2025;23(1):276. doi: 10.1186/s12964-025-02283-z 40495180 PMC12153189

[7] Wang Z, Mo H, He Z, Chen A, Cheng P. Extracellular vesicles as an emerging drug delivery system for cancer treatment: Current strategies and recent advances. Biomed Pharmacother. 2022;153:113480. doi: 10.1016/j.biopha.2022.113480 36076581

[8] Wang J, Yin B, Lian J, Wang X. Extracellular Vesicles as Drug Delivery System for Cancer Therapy. Pharmaceutics. 2024;16(8):1029. doi: 10.3390/pharmaceutics16081029 39204374 PMC11359799

[9] Squadrito ML, Cianciaruso C, Hansen SK, De Palma M. EVIR: chimeric receptors that enhance dendritic cell cross-dressing with tumor antigens. Nat Methods. 2018;15(3):183–6. doi: 10.1038/nmeth.4579 29355847 PMC5833950

[10] Dang XTT, Phung CD, Lim CMH, Jayasinghe MK, Ang J, Tran T, et al. Dendritic cell-targeted delivery of antigens using extracellular vesicles for anti-cancer immunotherapy. Cell Prolif. 2024;57(7):e13622. doi: 10.1111/cpr.13622 38509634 PMC11216926

[11] Schioppa T, Gaudenzi C, Zucchi G, Piserà A, Vahidi Y, Tiberio L, et al. Extracellular vesicles at the crossroad between cancer progression and immunotherapy: focus on dendritic cells. J Transl Med. 2024;22(1):691. doi: 10.1186/s12967-024-05457-4 39075551 PMC11288070

[12] Matsumoto A, Asuka M, Takahashi Y, Takakura Y. Antitumor immunity by small extracellular vesicles collected from activated dendritic cells through effective induction of cellular and humoral immune responses. Biomaterials. 2020;252:120112. doi: 10.1016/j.biomaterials.2020.120112 32422494

[13] Zhang X, Yuan X, Shi H, Wu L, Qian H, Xu W. Exosomes in cancer: small particle, big player. J Hematol Oncol. 2015;8:83. doi: 10.1186/s13045-015-0181-x 26156517 PMC4496882

[14] Miyanishi M, Tada K, Koike M, Uchiyama Y, Kitamura T, Nagata S. Identification of Tim4 as a phosphatidylserine receptor. Nature. 2007;450(7168):435–9. doi: 10.1038/nature06307 17960135

[15] Nakai W, Yoshida T, Diez D, Miyatake Y, Nishibu T, Imawaka N, et al. A novel affinity-based method for the isolation of highly purified extracellular vesicles. Sci Rep. 2016;6:33935. doi: 10.1038/srep33935 27659060 PMC5034288

[16] Yang J, Chen G, Wang R, Song C, Yi H. Navigating TAM receptor dynamics in tumour immunotherapy. Cancer Immunol Immunother. 2025;74(5):146. doi: 10.1007/s00262-024-03879-z 40088262 PMC11910493

[17] Rothlin CV, Carrera-Silva EA, Bosurgi L, Ghosh S. TAM receptor signaling in immune homeostasis. Annu Rev Immunol. 2015;33:355–91. doi: 10.1146/annurev-immunol-032414-112103 25594431 PMC4491918

[18] Lemke G, Rothlin CV. Immunobiology of the TAM receptors. Nat Rev Immunol. 2008;8(5):327–36. doi: 10.1038/nri2303 18421305 PMC2856445

[19] Seitz HM, Camenisch TD, Lemke G, Earp HS, Matsushima GK. Macrophages and dendritic cells use different Axl/Mertk/Tyro3 receptors in clearance of apoptotic cells. J Immunol. 2007;178(9):5635–42. doi: 10.4049/jimmunol.178.9.5635 17442946

[20] Geng K, Kumar S, Kimani SG, Kholodovych V, Kasikara C, Mizuno K, et al. Requirement of Gamma-Carboxyglutamic Acid Modification and Phosphatidylserine Binding for the Activation of Tyro3, Axl, and Mertk Receptors by Growth Arrest-Specific 6. Front Immunol. 2017;8:1521. doi: 10.3389/fimmu.2017.01521 29176978 PMC5686386

[21] Lemke G. Phosphatidylserine Is the Signal for TAM Receptors and Their Ligands. Trends Biochem Sci. 2017;42(9):738–48. doi: 10.1016/j.tibs.2017.06.004 28734578 PMC5600686

[22] Skotland T, Sagini K, Sandvig K, Llorente A. An emerging focus on lipids in extracellular vesicles. Adv Drug Deliv Rev. 2020;159:308–21. doi: 10.1016/j.addr.2020.03.002 32151658

[23] Yang JS, Kim JY, Lee JC, Moon MH. Investigation of lipidomic perturbations in oxidatively stressed subcellular organelles and exosomes by asymmetrical flow field-flow fractionation and nanoflow ultrahigh performance liquid chromatography-tandem mass spectrometry. Anal Chim Acta. 2019;1073:79–89. doi: 10.1016/j.aca.2019.04.069 31146839

[24] Dixon AS, Schwinn MK, Hall MP, Zimmerman K, Otto P, Lubben TH, et al. NanoLuc Complementation Reporter Optimized for Accurate Measurement of Protein Interactions in Cells. ACS Chem Biol. 2016;11(2):400–8. doi: 10.1021/acschembio.5b00753 26569370

[25] Kimani SG, Kumar S, Bansal N, Singh K, Kholodovych V, Comollo T, et al. Small molecule inhibitors block Gas6-inducible TAM activation and tumorigenicity. Sci Rep. 2017;7:43908. doi: 10.1038/srep43908 28272423 PMC5341070

[26] Heiring C, Dahlbäck B, Muller YA. Ligand recognition and homophilic interactions in Tyro3: structural insights into the Axl/Tyro3 receptor tyrosine kinase family. J Biol Chem. 2004;279(8):6952–8. doi: 10.1074/jbc.M311750200 14623883

[27] Shao H, Lauffenburger D, Wells A. Tyro3 carboxyl terminal region confers stability and contains the autophosphorylation sites. Biochem Biophys Res Commun. 2017;490(3):1074–9. doi: 10.1016/j.bbrc.2017.06.168 28668391 PMC5553559

[28] Rothlin CV, Ghosh S, Zuniga EI, Oldstone MBA, Lemke G. TAM receptors are pleiotropic inhibitors of the innate immune response. Cell. 2007;131(6):1124–36. doi: 10.1016/j.cell.2007.10.034 18083102

[29] Chan PY, Carrera Silva EA, De Kouchkovsky D, Joannas LD, Hao L, Hu D, et al. The TAM family receptor tyrosine kinase TYRO3 is a negative regulator of type 2 immunity. Science. 2016;352(6281):99–103. doi: 10.1126/science.aaf1358 27034374 PMC4935984

[30] Yanagihashi Y, Segawa K, Maeda R, Nabeshima Y-I, Nagata S. Mouse macrophages show different requirements for phosphatidylserine receptor Tim4 in efferocytosis. Proc Natl Acad Sci U S A. 2017;114(33):8800–5. doi: 10.1073/pnas.1705365114 28768810 PMC5565444

[31] Li T, Chiou B, Gilman CK, Luo R, Koshi T, Yu D, et al. A splicing isoform of GPR56 mediates microglial synaptic refinement via phosphatidylserine binding. EMBO J. 2020;39(16):e104136. doi: 10.15252/embj.2019104136 32452062 PMC7429740

[32] Mejia EM, Hatch GM. Mitochondrial phospholipids: role in mitochondrial function. J Bioenerg Biomembr. 2016;48(2):99–112. doi: 10.1007/s10863-015-9601-4 25627476

[33] Marzoog BA, Vlasova TI. Membrane lipids under norm and pathology. Eur J Clin Exp Med. 2021;19(1):59–75. doi: 10.15584/ejcem.2021.1.9

[34] D’Acunzo P, Argyrousi EK, Ungania JM, Kim Y, DeRosa S, Pawlik M, et al. Mitovesicles secreted into the extracellular space of brains with mitochondrial dysfunction impair synaptic plasticity. Mol Neurodegener. 2024;19(1):34. doi: 10.1186/s13024-024-00721-z 38616258 PMC11017499

[35] Joyce LR, Doran KS. Gram-positive bacterial membrane lipids at the host-pathogen interface. PLoS Pathog. 2023;19(1):e1011026. doi: 10.1371/journal.ppat.1011026 36602959 PMC9815629

[36] Joyce LR, Guan Z, Palmer KL. Streptococcus pneumoniae, S. pyogenes and S. agalactiae membrane phospholipid remodelling in response to human serum. Microbiology (Reading). 2021;167(5):001048. doi: 10.1099/mic.0.001048 33983874 PMC8290102

[37] Zhou Y, Wang Y, Chen H, Xu Y, Luo Y, Deng Y, et al. Immuno-oncology: are TAM receptors in glioblastoma friends or foes? Cell Commun Signal. 2021;19(1):11. doi: 10.1186/s12964-020-00694-8 33509214 PMC7841914

[38] Holtzhausen A, Harris W, Ubil E, Hunter DM, Zhao J, Zhang Y, et al. TAM Family Receptor Kinase Inhibition Reverses MDSC-Mediated Suppression and Augments Anti-PD-1 Therapy in Melanoma. Cancer Immunol Res. 2019;7(10):1672–86. doi: 10.1158/2326-6066.CIR-19-0008 31451482 PMC6943983

[39] Miao YR, Rankin EB, Giaccia AJ. Therapeutic targeting of the functionally elusive TAM receptor family. Nat Rev Drug Discov. 2024;23(3):201–17. doi: 10.1038/s41573-023-00846-8 38092952 PMC11335090

[40] Subramanian M, Hayes CD, Thome JJ, Thorp E, Matsushima GK, Herz J, et al. An AXL/LRP-1/RANBP9 complex mediates DC efferocytosis and antigen cross-presentation in vivo. J Clin Invest. 2014;124(3):1296–308. doi: 10.1172/JCI72051 24509082 PMC3934164

[41] Hafizi S, Gustafsson A, Stenhoff J, Dahlbäck B. The Ran binding protein RanBPM interacts with Axl and Sky receptor tyrosine kinases. Int J Biochem Cell Biol. 2005;37(11):2344–56. doi: 10.1016/j.biocel.2005.05.006 15964779

[42] Miyamoto Y, Torii T, Takada S, Ohno N, Saitoh Y, Nakamura K, et al. Involvement of the Tyro3 receptor and its intracellular partner Fyn signaling in Schwann cell myelination. Mol Biol Cell. 2015;26(19):3489–503. doi: 10.1091/mbc.E14-05-1020 26224309 PMC4591693

[43] Chesarino NM, Hach JC, Chen JL, Zaro BW, Rajaram MV, Turner J, et al. Chemoproteomics reveals Toll-like receptor fatty acylation. BMC Biol. 2014;12:91. doi: 10.1186/s12915-014-0091-3 25371237 PMC4240870

[44] Caraux A, Lu Q, Fernandez N, Riou S, Di Santo JP, Raulet DH, et al. Natural killer cell differentiation driven by Tyro3 receptor tyrosine kinases. Nat Immunol. 2006;7(7):747–54. doi: 10.1038/ni1353 16751775

[45] Peeters MJW, Dulkeviciute D, Draghi A, Ritter C, Rahbech A, Skadborg SK, et al. MERTK Acts as a Costimulatory Receptor on Human CD8+ T Cells. Cancer Immunol Res. 2019;7(9):1472–84. doi: 10.1158/2326-6066.CIR-18-0841 31266785

[46] Escudier B, Dorval T, Chaput N, André F, Caby M-P, Novault S, et al. Vaccination of metastatic melanoma patients with autologous dendritic cell (DC) derived-exosomes: results of thefirst phase I clinical trial. J Transl Med. 2005;3(1):10. doi: 10.1186/1479-5876-3-10 15740633 PMC554765

[47] Besse B, Charrier M, Lapierre V, Dansin E, Lantz O, Planchard D, et al. Dendritic cell-derived exosomes as maintenance immunotherapy after first line chemotherapy in NSCLC. Oncoimmunology. 2015;5(4):e1071008. doi: 10.1080/2162402X.2015.1071008 27141373 PMC4839329

[48] Yao Y, Fu C, Zhou L, Mi Q-S, Jiang A. DC-Derived Exosomes for Cancer Immunotherapy. Cancers (Basel). 2021;13(15):3667. doi: 10.3390/cancers13153667 34359569 PMC8345209

[49] Lin Y-C, Boone M, Meuris L, Lemmens I, Van Roy N, Soete A, et al. Genome dynamics of the human embryonic kidney 293 lineage in response to cell biology manipulations. Nat Commun. 2014;5:4767. doi: 10.1038/ncomms5767 25182477 PMC4166678

[50] Castle JC, Kreiter S, Diekmann J, Löwer M, van de Roemer N, de Graaf J, et al. Exploiting the mutanome for tumor vaccination. Cancer Res. 2012;72(5):1081–91. doi: 10.1158/0008-5472.CAN-11-3722 22237626

[51] Shen Z, Reznikoff G, Dranoff G, et al. Cloned Dendritic Cells Can Present Exogenous Antigens on Both M H C Class I and Class II Molecules’. J Immunol. 1997;158(6):2723–30. 9058806

[52] Kobayashi M, Kojima K, Murayama K, Amano Y, Koyama T, Ogama N, et al. MK-6, a novel not-α IL-2, elicits a potent antitumor activity by improving the effector to regulatory T cell balance. Cancer Sci. 2021;112(11):4478–89. doi: 10.1111/cas.15127 34545658 PMC8586658

[53] Güler-Gane G, Kidd S, Sridharan S, Vaughan TJ, Wilkinson TCI, Tigue NJ. Overcoming the Refractory Expression of Secreted Recombinant Proteins in Mammalian Cells through Modification of the Signal Peptide and Adjacent Amino Acids. PLoS One. 2016;11(5):e0155340. doi: 10.1371/journal.pone.0155340 27195765 PMC4873207

[54] Hall MP, Unch J, Binkowski BF, Valley MP, Butler BL, Wood MG, et al. Engineered luciferase reporter from a deep sea shrimp utilizing a novel imidazopyrazinone substrate. ACS Chem Biol. 2012;7(11):1848–57. doi: 10.1021/cb3002478 22894855 PMC3501149

[55] Matsui T, Osaki F, Hiragi S, Sakamaki Y, Fukuda M. ALIX and ceramide differentially control polarized small extracellular vesicle release from epithelial cells. EMBO Rep. 2021;22(5):e51475. doi: 10.15252/embr.202051475 33724661 PMC8097368

[56] Théry C, Amigorena S, Raposo G, Clayton A. Isolation and characterization of exosomes from cell culture supernatants and biological fluids. Curr Protoc Cell Biol. 2006;Chapter 3:Unit 3.22. doi: 10.1002/0471143030.cb0322s30 18228490

[57] Ishiai T, Subsomwong P, Narita K, Kawai N, Teng W, Suzuki S, et al. Extracellular vesicles of Pseudomonas aeruginosa downregulate pyruvate fermentation enzymes and inhibit the initial growth of Staphylococcus aureus. Curr Res Microb Sci. 2023;4:100190. doi: 10.1016/j.crmicr.2023.100190 37131486 PMC10149184

[58] Hikita T, Miyata M, Watanabe R, Oneyama C. Sensitive and rapid quantification of exosomes by fusing luciferase to exosome marker proteins. Sci Rep. 2018;8(1):14035. doi: 10.1038/s41598-018-32535-7 30232365 PMC6145919

