# Supplementary material for: Tyro3 facilitates cytoplasmic delivery of extracellular vesicle contents and antigen presentation via major histocompatibility complex class I in dendritic cells
Source: PLoS One. 2026 Aug 4;21(8):e0355054. doi: 10.1371/journal.pone.0355054 (PMC13436743; doi:10.1371/journal.pone.0355054)
Supplement: Supplementary file 1 [file pone.0355054.s011.docx]

**S1 Table**

| name | insert | C-terminal tag | vector type |
| --- | --- | --- | --- |
| pcDNA4-Sec-mTyro3-ECD-3×G4S-Myc-7×His | Mouse Tyro3  (aa 25–412) | Myc  7×His | mammalian expression |
| pcDNA4-Sec-mAxl-ECD-3×G4S-Myc-7×His | Mouse Axl  (aa 19–443) | Myc  7×His | mammalian expression |
| pcDNA4-Sec-mMertk-ECD-3×G4S-Myc-7×His | Mouse Mertk  (aa 23–497) | Myc  7×His | mammalian expression |
| pSIN-EF-mGas6-3×FLAG | Mouse Gas6  (aa1-674) | 3×FLAG | Lentiviral expression |
| pSIN-EF-mPros1-3×FLAG | Mouse Pros1  (aa1-675) | 3×FLAG | Lentiviral expression |
