# Supplementary material for: Tyro3 facilitates cytoplasmic delivery of extracellular vesicle contents and antigen presentation via major histocompatibility complex class I in dendritic cells
Source: PLoS One. 2026 Aug 4;21(8):e0355054. doi: 10.1371/journal.pone.0355054 (PMC13436743; doi:10.1371/journal.pone.0355054)
Supplement: Supplementary file 2 [file pone.0355054.s012.docx]

**S2 Table**

| **Primary antibodies** | | | | |
| --- | --- | --- | --- | --- |
| Target | Clone | Conjugate | Source | Application |
| mouse Dtk/Tyro3 | 109646 | PE | R&D Systems | FC, Extracellular |
| mouse Axl | MAXL8DS | PE | Invitrogen | FC, Extracellular |
| mouse Axl | 175128 | BV421 | BD Biosciences | FC, Intracellular |
| mouse Mertk | 2B10C42 | APC | BioLegend | FC, Extracellular |
| mouse CD8α | 53-6.7 | Pacific Blue | BioLegend | FC, Extracellular |
| mouse F4/80 | BM8 | APC, FITC | BioLegend | FC, Extracellular |
| mouse CD11c | N418 | PE, FITC | BioLegend | FC, Extracellular |
| mouse I-A/I-E | M5/114.15.2 | Pacific Blue | BioLegend | FC, Extracellular |
| human Tyro3 | 96201 | PE | R&D Systems | FC, Extracellular |
| human Axl | DS7HAXL | PE | Invitrogen | FC, Extracellular |
| human Mertk | 590H11G1E3 | PE | BioLegend | FC, Extracellular |
| HA tag | 16B12 | Alexa Fluor 488 | BioLegend | FC, Intracellular |
| FLAG tag | L5 | PerCP/Cy5.5 | BioLegend | FC, Intracellular |
| human Gas6 | D3A3G | - | Cell Signaling Technology | WB |
| human Pros1 | F-10 | - | Santa Cruz Biotechnology | WB |
| human CD63 | 3-13 | - | FUJIFILM Wako | WB |
| human Alix | 1A12 | - | Santa Cruz Biotechnology | WB |
| mouse Tyro3 | D38C6 | - | Cell Signaling Technology | WB |
| mouse GAPDH | 3H12 | HRP | MBL | WB |
| HA tag | HA.C5 | - | Invitrogen | IF |
| human Lamp-1 | H5G11 | - | Santa Cruz Biotechnology | IF |
| His tag | OGHis | HRP | MBL | POL |
| Myc tag | My3 | HRP | MBL | POL |
|  |  |  |  |  |
| **Secondary antibodies** | | | | |
| Target | Cat. No. | Conjugate | Source | Application |
| Rabbit IgG | 7074 | HRP | Cell Signaling Technology | WB |
| Mouse IgG | 7076 | HRP | Cell Signaling Technology | WB |
| Mouse IgG | A-11005 | Alexa Fluor 594 | Invitrogen | IF |
| Rabbit IgG | A28181 | Alexa Fluor 647 | Invitrogen | IF |

**Abbreviations:** FC, flow cytometry; WB, western blot; IF, immunofluorescence; POL, phospholipid overlay assay
