# Supplementary material for: Tyro3 facilitates cytoplasmic delivery of extracellular vesicle contents and antigen presentation via major histocompatibility complex class I in dendritic cells
Source: PLoS One. 2026 Aug 4;21(8):e0355054. doi: 10.1371/journal.pone.0355054 (PMC13436743; doi:10.1371/journal.pone.0355054)
Supplement: Supplementary file 3 [file pone.0355054.s015.docx]

Suzie Chen

Academic Editor, PLOS One

RE: Ms No.: PONE-D-26-03793

Dear. Prof. Chen

We appreciate the Reviewer's comments for our manuscript entitled " Tyro3 facilitates cytoplasmic delivery of extracellular vesicle contents and antigen presentation via major histocompatibility

complex class I in dendritic cells"

(PONE-D-26-03793)

First of all, We would like to thank the Academic Editor and the two reviewers for their helpful comments. We have carefully considered all comments and revised the manuscript accordingly. We thus feel that the attached revised manuscript is complete in this regard. We provide point-by-point responses to the journal requirements, the Academic Editor’s comments, and the reviewers’ comments.

**Point by point Reply for the Journal Requirements：**

Requirement #1), PLOS ONE style requirements.

"Please ensure that your manuscript meets PLOS ONE's style requirements, including those for file naming."

We have revised the manuscript to comply with PLOS ONE's style requirements, including the requirements for file naming. The revised manuscript, the marked-up manuscript, and this response letter have been prepared and uploaded according to the journal's instructions.

Requirement #2), Original images for blot and gel data.

"PLOS ONE now requires that authors provide the original uncropped and unadjusted images underlying all blot or gel results reported in a submission’s figures or Supporting Information files."

We have provided the original uncropped and unadjusted images underlying the blot and phospholipid overlay data reported in the manuscript and Supporting Information files. These raw image data are included as Figure S8. We have also ensured that the revised figures comply with PLOS ONE’s requirements for blot/gel reporting and figure preparation. No raw blot/gel images are unavailable.

Requirement #3), Funding information.

"We note that the grant information you provided in the ‘Funding Information’ and ‘Financial Disclosure’ sections do not match. When you resubmit, please ensure that you provide the correct grant numbers for the awards you received for your study in the ‘Funding Information’ section."

We appreciate the editorial office’s careful check. We have resolved the discrepancy between the Funding Information and Financial Disclosure sections by correcting the grant information in both the submission system and the Funding Information section of the revised manuscript (Lines 689-697).

Requirement #4), Funding information.

"Please state what role the funders took in the study. If the funders had no role, please state: 'The funders had no role in study design, data collection and analysis, decision to publish, or preparation of the manuscript.'"

We have included the requested Role of Funder statement in the cover letter. The revised Funding information section of the manuscript also includes the following statement: "The funders had no role in study design, data collection and analysis, decision to publish, or preparation of the manuscript."

Requirement #5), Ethics statement.

"Please include your full ethics statement in the ‘Methods’ section of your manuscript file."

We have included a full ethics statement in the Methods section of the revised manuscript. The statement includes the full name of the animal experiment committee that approved the study, the approval number, and the animal care and euthanasia procedures. We also clarified that no human participants, primary human samples, or identifiable human data were used in this study and that informed consent was therefore not applicable.

Requirement #6), Citation of previously published works.

"If the reviewer comments include a recommendation to cite specific previously published works, please review and evaluate these publications to determine whether they are relevant and should be cited."

This comment is related to comment #2 from Reviewer 1. We have reviewed the previously published work recommended by the reviewer and evaluated its relevance to our study. Because Rothlin et al. (Nat Rev Immunol, 2015) provides important background on the roles of TAM receptors in immune regulation, we considered this work relevant and have cited it in the revised manuscript.

**Additional Editor Comments:**

"Please read the suggestions and comments from each reviewers and answer each comments specifically with details. The reviewers took a lot of time to review the manuscript please read their comments carefully and respond properly."

We thank the Academic Editor for this comment. We have carefully read all comments from the reviewers and have addressed each point in detail. Point-by-point responses to all reviewer comments are provided below, and the corresponding changes have been incorporated into the revised manuscript where appropriate.

**Point by point Reply for the comments of Reviewer #1:**

Comment #1) Insufficient information in the manuscript and figure legends.

"The manuscript generally lacks important information that is needed to understand it. Figure legends are usually too brief, and figures are difficult to understand. Key materials are not well-explained. Need an explanation for each material presented."

We agree that several key materials and experimental tools were not sufficiently explained in the original manuscript. We have therefore revised the manuscript and figure legends extensively. In the revised manuscript, we added a detailed description of the CD63-based sEV tools in the “Preparation of sEVs” section (Lines 548–553). We also clarified the purpose of each CD63-based sEV tool in the Results sections, including CD63-NLuc-sEVs (Lines 104–105), CD63-HiBiT-sEVs (Lines 221–224), CD63-dClover2-sEVs (Lines 233–235), and CD63-OVA-sEVs (Lines 311–314). We further defined +Gas6-sEVs and +Pros1-sEVs as sEVs preincubated with Gas6 or Pros1, respectively (Lines 119–122). We also substantially expanded the legends for Figures 1–4 (Lines 142-160, 201-219, 278-295, and 324-351) to include the experimental design, cell lines, assay methods, quantification procedures, fluorescence colors, scale bars, and sample numbers. We also revised the figure titles to better reflect the contents of each figure.

Comment #2), TAM receptor expression in DC2.4 cells.

" Please discuss or justify the absence of detectable TAM receptor expression in this dendritic cell line (DC2.4)."

In the revised Discussion, we now explicitly discuss this point (Line 393-405). As the reviewer noted, TAM receptors are important regulators of immune homeostasis in dendritic cells and other antigen-presenting cells. However, we did not detect endogenous surface expression of Tyro3, Axl, or Mertk in parental DC2.4 cells by flow cytometry under our assay conditions. Previous studies have shown that the expression patterns of individual TAM receptors vary depending on the DC subset, differentiation conditions, activation status, inflammatory context, and detection method. We also note that DC2.4 is an immortalized dendritic cell line and may not fully recapitulate the TAM receptor expression patterns of primary DC populations. Accordingly, we used DC2.4 cells individually expressing exogenous TAM receptors to evaluate the receptor-specific contribution of Tyro3, Axl, and Mertk to sEV association and internalization.

Comment #3) Rationale for the use of DC2.4 and HeLa cells.

"Rationale for switching between cell type: the study switches between DC2.4 cells and HeLa cells for different analyses, but the rationale for this choice is not clearly explained."

In the originally submitted manuscript, HeLa cells were used for confocal microscopy analyses because their flat morphology is suitable for obtaining clear intracellular images. However, we agree with the reviewer that the use of different cell types for different analyses was insufficiently justified and could make the interpretation of the results difficult. To address this concern, we repeated the HeLa-based confocal microscopy experiments using DC2.4-derived dendritic cell lines, which are the main cellular model used in this study. Accordingly, we replaced the previous HeLa cell data with new DC2.4 cell data in the revised manuscript. The revised data are now shown in Figure 3B and Figure 4E. We also revised the corresponding Results sections and figure legends to reflect these changes.

Comment #4), Visualization of sEV internalization in dendritic cells.

"Given the importance of EV uptake in antigen presentation in dendritic cells, visualization sEV internalization in dendritic cells would be informative."

We thank the reviewer for this helpful suggestion. In the revised manuscript, we visualized the intracellular localization of CD63-dClover2-sEV-derived signals in DC2.4 cells expressing Tyro3 or its ectodomain deletion mutants. The schematic illustration of the Tyro3 mutants, which was previously shown as Figure 2F, has been reorganized and is now shown as Figure 3A. The new confocal microscopy data obtained using DC2.4-derived cells are shown in Figure 3B. The corresponding Results section has been revised accordingly (Lines 268–276), and the figure legend has also been updated.

**Point by point Reply for the comments of Reviewer #2:**

Comment #1), Improvement of figure readability.

"Improvements in all the figures are required to improve their readability as it is blurry."

In the revised manuscript, we have improved the readability of all figures by increasing the font size and reorganizing the layout of the figure panels. We also prepared and uploaded higher-resolution figure files, within the file-size limits accepted by the PLOS ONE submission system.

Comment 2), Reorganization of sEV characterization data.

"Gas-6, Pros-1, and CD63 expressions in these sEVs in figure 2 should be present in figure 1 as chronologically it makes more sense after Figure 1B."

We thank the reviewer for this helpful suggestion. As suggested, we have reorganized the figures and moved the western blotting data for Gas6 and Pros1 in the sEV preparations from the original Figure 2A to the revised Figure 1D. This revision allows the characterization of the sEV preparations to be presented before the subsequent binding and cellular association assays. We have also revised the corresponding text in the Results section accordingly (Lines 114–119).

Comment 3), Evaluation of sEV preparation by electron microscopy.

"The purity of the isolation method of these sEV’s must be evaluated using electron micrograph."

We thank the reviewer for this important suggestion. In response, we performed transmission electron microscopy analysis of the sEV preparations isolated from Expi293F cells. The electron micrograph has been added as Figure 1C in the revised manuscript. We also revised the corresponding Results section to describe the TEM-based evaluation of the sEV preparations (Lines 112–114) and added the experimental details to the Materials and Methods section (Lines 565–567).

Comment #4), Pull-down assay.

"There should be figure 1 describing the readout of figure 1c to help the reader conceptualize the data."

As requested, we have added a schematic diagram of the pull-down assay workflow to the revised Figure 1E. This schematic illustrates how TAM receptor ectodomains were immobilized on beads, incubated with CD63-NLuc-sEVs, washed, and then evaluated by NanoLuc luciferase activity.

Comment #5), Background control in the pull-down assay.

"Figure 1c there should be a plain supernatant that has gone through the same process as the sEVs in figure 1 to evaluate the background signal that has no sEVs."

We thank the reviewer for this important suggestion. We agree that a processed no-sEV supernatant would be an appropriate control for estimating the absolute background signal in this assay. In the present experiment, NLuc activity was used as a readout for CD63-NLuc-labeled sEVs bound to individual TAM-ECDs. CD63-NLuc-sEVs, +Gas6-sEVs, and +Pros1-sEVs were applied as equivalent NLuc inputs and subjected to the same pull-down, washing, and detection procedures. Therefore, Fig 1F represents a relative comparison of CD63-NLuc-labeled sEV binding to individual TAM-ECDs under different ligand conditions. To avoid overinterpretation, we have clarified in the revised Results section that this assay was designed to evaluate relative TAM-ECD-associated CD63-NLuc-sEV signals under matched input conditions (Lines 123–127).

Comment #6), Sample sizes in figure legends.

"Sample sizes for all experiments must be explicitly stated in the figure legend."

In the revised manuscript, we have added the sample sizes to the relevant figure legends for Figure 1–4 (Lines 142-160, 201-219, 278-295, and 324-351).

Comment #7), Phosphatase treatment of phospholipids.

"For figure 1e and 1f phosphatase should be added to the phospholipids at varying concentrations to evaluate if the binding would be abrogated."

We thank the reviewer for this suggestion. In this study, we did not perform phosphatase treatment of the phospholipids. The phospholipid overlay assay was used to evaluate the relative binding of TAM ligands and TAM-ECDs to individual phospholipids under defined conditions. We agree that additional competition or blocking experiments, such as masking phosphatidylserine with annexin V, would further support the specificity of the interaction. We have noted this point as a limitation and an important subject for future study.

Comment #8), Use of HeLa cells in Figure 2.

"In figure 2, why weren’t the results of HeLa cells shown as it was mentioned that both DC2.4 and HeLa cells were used. The results of this must be shown."

We appreciate the reviewer’s careful reading. The experiments shown in Figure 2 were performed using DC2.4 cells, not HeLa cells. In the original manuscript, the sentence “We assessed the binding and uptake of sEVs by TAM-expressing cells using DC2.4 and HeLa cells” (original Lines 153–154) was misleading, as it could be interpreted to mean that the sEV association and uptake experiments were performed in both DC2.4 and HeLa cells. In the revised manuscript, we have corrected this description to clearly indicate that the Figure 2 experiments were performed using TAM receptor-expressing DC2.4 cells (Lines 180–182).

Comment #9), Validation in another dendritic cell line.

"All figure 2 data and data using one dendritic cell line must be validated in another dendritic cell line. The authors mentioned that they did similar experiments in HeLa cells; this must be shown."

We thank the reviewer for this important comment. We agree that validation in another dendritic cell line would provide additional support for the findings shown in Figure 2. However, in the present revision, we did not perform the full set of Figure 2 experiments in another dendritic cell line. Therefore, we have revised the manuscript to clearly state that the Figure 2 experiments were performed using TAM receptor-expressing DC2.4 cells and have limited our interpretation accordingly. We agree that validation in additional dendritic cell models remains an important future direction.

As also described in our response to Comment #8 from Reviewer 2, the original statement referring to both DC2.4 and HeLa cells was misleading. We have corrected this description in the revised manuscript.

Comment #10), Functional evaluation of Gas6-dependent sEV association and uptake.

"Figures 2B and 2C should have sEVs negative for Gas6 and Pros1 (negative control)."

We thank the reviewer for this important suggestion. We agree that Gas6/Pros1-negative sEVs would be a useful negative control. However, generating such sEVs from Expi293F producer cells would require additional genetic manipulation and careful validation to confirm that sEV production, size distribution, and CD63-reporter content are not substantially affected. Therefore, in the present revision, we performed a functional inhibition experiment using RU-302, a Gas6–TAM interaction inhibitor. Because the HiBiT-LgBiT system more directly evaluates sEV internalization than the CD63-NLuc-sEV system, we performed this additional experiment using CD63-HiBiT-sEVs and LgBiT-expressing DC2.4 cell lines. The new data are shown in Figure S2 and described in the revised Results section (Lines 250–255) . RU-302 significantly reduced HiBiT–LgBiT luminescence in LgBiT-DC2.4Tyro3 cells, supporting the involvement of the Gas6–Tyro3 axis in sEV uptake by Tyro3-expressing DC2.4 cells. We have also avoided overinterpreting these results, because this pharmacological inhibition experiment does not exclude possible contributions from Pros1 or other mechanisms.

Comment #11), Functional inhibition of Gas6–TAM-mediated sEV uptake.

"Figure 2D and 2E should have the EVs negative for Gas6 and Pros1 (negative control)."

This comment is related to Comment #10 from Reviewer 2. To address this point in the present revision, we performed a functional inhibition experiment using RU-302 and CD63-dClover2-sEVs. The new data are shown in Figure 2E and described in the revised Results section (Lines 240–250). We also added the corresponding experimental details to the Materials and Methods section (Lines 627–631).

Comment #12), Functional blockade of Gas6–TAM receptor-mediated sEV uptake.

"Figure 2 should have antibody blocking assays/competition assay done to see how sEV internalization differs when the TAM receptors studied are blocked."

This comment is related to Comment #10 and Comment #11 from Reviewer 2. We agree that antibody-blocking or competition assays would provide further evidence supporting the involvement of TAM receptors in sEV internalization. However, to the best of our knowledge, suitable and validated blocking antibodies against the extracellular domains of the TAM receptors used in this study are not readily available. In addition, competition experiments using recombinant Gas6- or Pros1-derived Gla-domain proteins would require careful optimization and validation. Therefore, in the present revision, we performed a functional inhibition experiment using RU-302. Direct receptor-blocking or ligand-competition experiments remain an important subject for future studies.

Comment #13), Validation of Figure 3 using DC2.4 and another dendritic cell lines.

"Figure 3 should be validated in another cell line such as DC2.4 and another dendritic cell line."

This comment is related to Comment #4 from Reviewer 1 and Comment #9 from Reviewer 2. As also described in our response to Comment #4 from Reviewer 1, we visualized the intracellular localization of CD63-dClover2-sEV-derived signals in DC2.4 cells expressing Tyro3 or its ectodomain deletion mutants, as shown in Figure 3B. The corresponding Results section has been revised accordingly (Lines 268–276), and the figure legend has also been updated.

We agree that further validation in another dendritic cell line would provide additional support for these findings. As also described in our response to Comment #9 from Reviewer 2, we focused on validation using DC2.4 cells, which were used as the dendritic cell model in this study. We have revised the manuscript to clearly state that these experiments were performed using DC2.4 cells expressing Tyro3 and have limited our interpretation accordingly. Validation in additional dendritic cell models remains an important subject for future studies.

Comment #14), IG2c-specific blocking or competition assay.

"Figure 3 should have antibody blocking assays/competition assay done to see how sEV internalization differs when IG2c Tyro3 domain is blocked."

We thank the reviewer for this important suggestion. We agree that an antibody blocking or competition assay targeting the IG2c region of Tyro3 would provide a more direct test of the role of this region in sEV internalization. However, a validated blocking antibody that specifically inhibits the IG2c region of Tyro3 without affecting overall receptor conformation is not available. In this study, we evaluated the role of the IG2c-containing region using Tyro3 ectodomain deletion mutants. These results suggest that this region contributes to Tyro3-mediated sEV association and/or internalization, but they do not directly demonstrate ligand binding to the IG2c region. Future biochemical competition assays using recombinant Tyro3 IG2c-containing fragments and LG-domain-containing fragments of Gas6 or Pros1 would help clarify whether this region directly contributes to ligand–Tyro3 binding. We therefore consider this an important subject for future studies.

Comment #15), Gas6/Pros1-negative sEV controls in original Figure 3.

"Figure 3 should have sEVs negative for Gas6 and Pros1 (negative control)."

We agree that Gas6/Pros1-negative sEVs would be a useful negative control to evaluate the contribution of endogenous Gas6 and Pros1 associated with sEVs. As also described in our response to Comment #10 from Reviewer 2, generating Gas6/Pros1-negative sEVs from Expi293F producer cells would require additional genetic manipulation and careful validation. Therefore, in the revised manuscript, we performed a functional inhibition experiment using RU-302, as an alternative approach. Using the CD63-dClover2-sEV association assay, we found that RU-302 reduced sEV-derived dClover2 MFI in cells expressing intact Tyro3 or the ΔIG mutant (revised Figure 3C). These results are described in the revised Results section (Lines 297–305) and support the involvement of the Gas6–Tyro3 axis in sEV association and/or uptake through the IG2c-containing region. We acknowledge that this experiment does not fully replace the use of Gas6/Pros1-negative sEVs, particularly for evaluating the possible contribution of Pros1. We consider the generation and validation of Gas6/Pros1-negative sEVs an important subject for future study.

Comment #16), Blocking and competition assays for TAM-dependent sEV uptake and MHC-I-restricted OT-I activation.

"Figure 4 should have antibody blocking assays/competition assay done to see how sEV internalization differs when the TAM receptors and/or MHC I are blocked and how it affects activation of CTLs. This should be done with the negative control of sEVs negative for Gas6 and Pros1 as well as sEVs with Gas6 and Pros1. These sEVs should be utilized for both in vitro and in vivo studies."

We thank the reviewer for this important suggestion. This comment is related to Comment #10, Comment #11, Comment #12, and Comment #15 from Reviewer #2. As described above, in the present revision we performed functional inhibition experiments using RU-302 to evaluate the contribution of the Gas6–TAM interaction to sEV association and internalization. These additional experiments support the involvement of the Gas6–Tyro3 axis in sEV uptake by Tyro3-expressing DC2.4 cells. We agree that extending these blocking or competition approaches to the CD63-OVA-sEV/OT-I cross-presentation assay. However, these experiments would require additional optimization and validation, particularly for generating Gas6/Pros1-defined sEV preparations. Therefore, we have limited our interpretation accordingly and have clarified that the Figure 4 in vitro data demonstrate enhanced cross-presentation of sEV-delivered antigen in the DC2.4-derived Tyro3-expressing cell system. Further blocking/competition experiments and validation using Gas6/Pros1-defined sEV preparations remain important subjects for future studies.

Comment #17), Tumor progression

"Tumor progression data from figure 4F and 4G should be shown."

In the revised manuscript, we have added the B16F10 tumor growth curve to Figure 4G. This graph shows the tumor progression in the individual mice used for the analysis of splenic dendritic cells shown in Figure 4H.

Comment #18), Validation of Figure 4 *in vitro* studies in another dendritic cell line.

"Figure 4 the *in vitro* studies should be validated using more than one dendritic cell line."

As also described in our response to Comment #9 from Reviewer 2. We agree that validation in an additional dendritic cell line, or in primary dendritic cells, would further strengthen the in vitro findings shown in Figure 4, particularly the cross-presentation assay. In the present study, the Figure 4 *in vitro* experiments were performed using DC2.4-derived cells to compare intact Tyro3 and its intracellular-domain mutants in the same cellular background. We did not perform validation in another dendritic cell line in the present revision. Accordingly, we have clarified that these experiments were performed using DC2.4-derived cells and have limited our interpretation to this experimental system. We consider validation in additional dendritic cell models and/or primary dendritic cells to be an important subject for future studies.

Comment #19), Addition of a schematic model.

"A figure describing the mechanism described in this manuscript of Tyro-3 mediated uptake of sEVs must be shown."

In the revised manuscript, we added a schematic model as Figure S7 to summarize the proposed mechanism of Tyro3-mediated sEV uptake and antigen cross-presentation described in this study. This figure illustrates that sEVs interact with Tyro3 through TAM ligands, such as Gas6 and/or Pros1, are internalized by Tyro3-expressing dendritic cells, and localize near Lamp-1-positive endolysosomal compartments. The model also summarizes the proposed contribution of the IG2c-containing extracellular region to sEV association and the TAPD-containing intracellular region to efficient cross-presentation of sEV-delivered antigens via MHC-I.

Comment #20), Clarification of mouse sex in the *in vivo* experiment.

"It must be explicitly stated for the *in vivo* experiment that female mice were used in the figure legend. Similar studies must be done using male mice to ensure no sex differences as of figure 4F and 4G."

We thank the reviewer for this important comment. In the revised manuscript, we explicitly stated in the Figure 4 legend that female mice were used in the in vivo experiment (Line 340). The Methods section also states that seven-week-old female C57BL/6 mice were used for subcutaneous inoculation of B16F10 cells (Line 506). We agree that studies using male mice would be important to evaluate possible sex-dependent differences. However, we have not performed additional experiments using male mice in the present revision. Therefore, we have limited our interpretation to the experimental conditions used in this study and do not conclude that the observed increase in Tyro3-positive splenic dendritic cells is independent of sex. We consider the evaluation of possible sex-dependent differences to be an important subject for future studies.

Comment #21), Description of the origin and sex information of the cells used in this study.

"The origin from which the cells used in this study came form must be described in detail including the sex."

We have revised the Materials and Methods section to provide more detailed information on the origins of the cells used in this study, including sex information when available (Line 466-477).

**Note to the Editor:**

We would like to inform the editorial office that two authors, Hitomi Kosai and Noriko Ishida, have been added to the revised manuscript. They contributed to the experiments and/or data analysis included in the revised manuscript and meet the authorship criteria. All authors, including the newly added authors, have reviewed and approved the revised manuscript and agreed to its submission to PLOS ONE.

**Additional Changes in the Revised Manuscript**

In addition to the point-by-point revisions described above, we carefully considered the overall comments from Reviewer 1 and Reviewer 2, which indicated that additional background information, experimental rationale, and logical connections would help readers better understand the manuscript. Accordingly, we made additional revisions to the Abstract, Introduction, Results, Materials and Methods, Discussion, Conclusions, References, and the supplementary figures and their legends to improve the clarity and completeness of the manuscript. These additional changes, as well as the revisions corresponding to the responses above, are highlighted in yellow in the revised manuscript.

Sincerely,

Takashi Koyama

Division of Tumor Immunology,

Miyagi Cancer Center Research Institute

47-1 Nodayama, Medeshima-Shiode,

Natori, Miyagi 981-1293, Japan

Email: takashi-koyama@miyagi-pho.jp

Tel: +81-22-384-3151
