# Supplementary material for: Tyro3 facilitates cytoplasmic delivery of extracellular vesicle contents and antigen presentation via major histocompatibility complex class I in dendritic cells
Source: PLoS One. 2026 Aug 4;21(8):e0355054. doi: 10.1371/journal.pone.0355054 (PMC13436743; doi:10.1371/journal.pone.0355054)
Supplement: Peer review history

# Peer review: Decision Letter 0

**DOI:** 10.1371/journal.pone.0355054.r001

**Authors:** Suzie Chen

9 Mar 2026

PONE-D-26-03793

Tyro3 facilitates cytoplasmic delivery of extracellular vesicle contents and antigen presentation via major histocompatibility complex class I in dendritic cells

PLOS One

Dear Dr. Koyama,

Thank you for submitting your manuscript to PLOS ONE. After careful consideration, we feel that it has merit but does not fully meet PLOS ONE’s publication criteria as it currently stands. Therefore, we invite you to submit a revised version of the manuscript that addresses the points raised during the review process.

Please submit your revised manuscript by Apr 23 2026 11:59PM. If you will need more time than this to complete your revisions, please reply to this message or contact the journal office at plosone@plos.org. When you're ready to submit your revision, log on to https://www.editorialmanager.com/pone/ and select the 'Submissions Needing Revision' folder to locate your manuscript file.

Please follow the instruction of the journal and respond to the comments from each reviewer with precise answers for all comments.

Please include the following items when submitting your revised manuscript:

- A letter that responds to each point raised by the academic editor and reviewer(s). You should upload this letter as a separate file labeled 'Response to Reviewers'.
- A marked-up copy of your manuscript that highlights changes made to the original version. You should upload this as a separate file labeled 'Revised Manuscript with Track Changes'.
- An unmarked version of your revised paper without tracked changes. You should upload this as a separate file labeled 'Manuscript'.

If you would like to make changes to your financial disclosure, please include your updated statement in your cover letter. Guidelines for resubmitting your figure files are available below the reviewer comments at the end of this letter.

If applicable, we recommend that you deposit your laboratory protocols in protocols.io to enhance the reproducibility of your results. Protocols.io assigns your protocol its own identifier (DOI) so that it can be cited independently in the future. For instructions see: https://journals.plos.org/plosone/s/submission-guidelines#loc-laboratory-protocols. Additionally, PLOS ONE offers an option for publishing peer-reviewed Lab Protocol articles, which describe protocols hosted on protocols.io. Read more information on sharing protocols at https://plos.org/protocols?utm_medium=editorial-email&utm_source=authorletters&utm_campaign=protocols.

We look forward to receiving your revised manuscript.

Kind regards,

Suzie Chen

Academic Editor

PLOS One

Journal Requirements:

When submitting your revision, we need you to address these additional requirements.

1.Please ensure that your manuscript meets PLOS ONE's style requirements, including those for file naming. The PLOS ONE style templates can be found at

https://journals.plos.org/plosone/s/file?id=wjVg/PLOSOne_formatting_sample_main_body.pdf and

https://journals.plos.org/plosone/s/file?id=ba62/PLOSOne_formatting_sample_title_authors_affiliations.pdf

2. PLOS ONE now requires that authors provide the original uncropped and unadjusted images underlying all blot or gel results reported in a submission’s figures or Supporting Information files. This policy and the journal’s other requirements for blot/gel reporting and figure preparation are described in detail at https://journals.plos.org/plosone/s/figures#loc-blot-and-gel-reporting-requirements and https://journals.plos.org/plosone/s/figures#loc-preparing-figures-from-image-files. When you submit your revised manuscript, please ensure that your figures adhere fully to these guidelines and provide the original underlying images for all blot or gel data reported in your submission. See the following link for instructions on providing the original image data: https://journals.plos.org/plosone/s/figures#loc-original-images-for-blots-and-gels.

In your cover letter, please note whether your blot/gel image data are in Supporting Information or posted at a public data repository, provide the repository URL if relevant, and provide specific details as to which raw blot/gel images, if any, are not available. Email us at plosone@plos.org if you have any questions.

3. We note that the grant information you provided in the ‘Funding Information’ and ‘Financial Disclosure’ sections do not match.

When you resubmit, please ensure that you provide the correct grant numbers for the awards you received for your study in the ‘Funding Information’ section.

4. Thank you for stating the following financial disclosure:

“This work was supported by Grants-in-Aid for Scientific Research (B) Grant Numbers JP23K24194 and JP22H02933, Scientific Research (C) Grant Number JP24K12661, and Early-Career Scientists Grant Number JP20K17766, from the Japan Society for the Promotion of Science (JSPS), Grants-in-Aid from Japan Science and Technology Agency (JST) CREST, Japan Science and Technology Agency Grant Numbers JPMJCR17H4, and Japan Agency for Medical Research and Development Grant Number 23ym0126101h0001.”

Please state what role the funders took in the study.  If the funders had no role, please state: "The funders had no role in study design, data collection and analysis, decision to publish, or preparation of the manuscript."

If this statement is not correct you must amend it as needed.

Please include this amended Role of Funder statement in your cover letter; we will change the online submission form on your behalf.

5. Please include your full ethics statement in the ‘Methods’ section of your manuscript file. In your statement, please include the full name of the IRB or ethics committee who approved or waived your study, as well as whether or not you obtained informed written or verbal consent. If consent was waived for your study, please include this information in your statement as well.

6. If the reviewer comments include a recommendation to cite specific previously published works, please review and evaluate these publications to determine whether they are relevant and should be cited. There is no requirement to cite these works unless the editor has indicated otherwise.

Additional Editor Comments:

Please read the suggestions and comments from each reviewers and answer each comments specifically with details. The reviewers took a lot of time to review the manuscript please read their comments carefully and respond properly.

[Note: HTML markup is below. Please do not edit.]

Reviewer's Responses to Questions

**Comments to the Author**

1. Is the manuscript technically sound, and do the data support the conclusions?

The manuscript must describe a technically sound piece of scientific research with data that supports the conclusions. Experiments must have been conducted rigorously, with appropriate controls, replication, and sample sizes. The conclusions must be drawn appropriately based on the data presented.

Reviewer #1: Yes

Reviewer #2: Partly

**********

2. Has the statistical analysis been performed appropriately and rigorously?

Reviewer #1: Yes

Reviewer #2: Yes

**********

3. Have the authors made all data underlying the findings in their manuscript fully available?

The PLOS Data policy requires authors to make all data underlying the findings described in their manuscript fully available without restriction, with rare exception (please refer to the Data Availability Statement in the manuscript PDF file). The data should be provided as part of the manuscript or its supporting information, or deposited to a public repository. For example, in addition to summary statistics, the data points behind means, medians and variance measures should be available. If there are restrictions on publicly sharing data—e.g. participant privacy or use of data from a third party—those must be specified.

Reviewer #1: Yes

Reviewer #2: Yes

**********

4. Is the manuscript presented in an intelligible fashion and written in standard English?

PLOS ONE does not copyedit accepted manuscripts, so the language in submitted articles must be clear, correct, and unambiguous. Any typographical or grammatical errors should be corrected at revision, so please note any specific errors here.

Reviewer #1: No

Reviewer #2: Yes

**********

5. Review Comments to the Author

Please use the space provided to explain your answers to the questions above. You may also include additional comments for the author, including concerns about dual publication, research ethics, or publication ethics. (Please upload your review as an attachment if it exceeds 20,000 characters)

Reviewer #1: Comments for Koyama et al to Plos One

This paper presents potential EV engineering for antigen presentation. TAM binds to GAS6 and Pros1 that are coated on the surface of EVs. GAS6 or Pros1 would not be present enough on the sEVs, and thus they supplemented it. GAS6 and Pros1 bind to PS on the EV membrane. The authors then successfully engineered EV uptake. The manuscript generally lacks important information that is needed to understand it. Figure legends are usually too brief, and figures are difficult to understand.

Key materials are not well-explained. For example, CD53-OVA-sEV is not explained and is not included in the cited reference (Engineered luciferase reporter from a deep-sea shrimp utilizing a novel imidazopyrazinone substrate). What is that? Need an explanation for each material presented.

Line 154: Flow cytometry analysis revealed no detectable endogenous TAM receptor expression in DC2.4 cells. This observation is unexpected, as dendritic cells are generally reported to express TAM family receptors, particularly MERTK and inducible AXL, which play key roles in immune regulation (Rothlin et al., Nat Rev Immunol, 2015). Please discuss or justify the absence of detectable TAM receptor expression in this dendritic cell line.

Rationale for switching between cell type: the study switches between DC2.4 cells and HeLa cells for different analyses, but the rationale for this choice is not clearly explained. In Figure 3, microscopic observations of the internalization and intracellular localization of CD63-dClover2-sEVs were performed in HeLa cells. Given the importance of EV uptake in antigen presentation in dendritic cells, visualization sEV internalization in dendritic cells would be informative.

Reviewer #2: Koyama et al in this manuscript describes the role of Tyro3 as a receptor on dendritic cells that mediates the uptake of sEVs and allows antigen cross-presentation in dendritic cells. Gas6 or Pros1 found on sEVs acts as a bridge between sEVs and Tyro3 on dendritic cells that leads to efficient internalization and requires the IG2c domain of its extracellular region. The authors narrowed this region to a 46-amino acid intracellular region of Tyro3 that designated it as the Tyro3 antigen presentation-related domain (TAPD). This manuscript adds a lot of value; however, to enhance the manuscript, the authors should make the following revisions.

-Improvements in all the figures are required to improve their readability as it is blurry.

-Gas-6, Pros-1, and CD63 expressions in these sEVs in figure 2 should be present in figure 1 as chronologically it makes more sense after Figure 1B.

-The purity of the isolation method of these sEV’s must be evaluated using electron micrograph.

-There should be figure 1 describing the readout of figure 1c to help the reader conceptualize the data.

-Figure 1c there should be a plain supernatant that has gone through the same process as the sEVs in figure 1 to evaluate the background signal that has no sEVs.

-Sample sizes for all experiments must be explicitly stated in the figure legend.

-For figure 1e and 1f phosphatase should be added to the phospholipids at varying concentrations to evaluate if the binding would be abrogated.

-In figure 2, why weren’t the results of HeLa cells shown as it was mentioned that both DC2.4 and HeLa cells were used. The results of this must be shown.

-All figure 2 data and data using one dendritic cell line must be validated in another dendritic cell line. The authors mentioned that they did similar experiments in HeLa cells; this must be shown.

-Figures 2B and 2C should have sEVs negative for Gas6 and Pros1 (negative control).

-Figure 2D and 2E should have the EVs negative for Gas6 and Pros1 (negative control).

-Figure 2 should have antibody blocking assays/competition assay done to see how sEV internalization differs when the TAM receptors studied are blocked.

-Figure 3 should be validated in another cell line such as DC2.4 and another dendritic cell line.

-Figure 3 should have antibody blocking assays/competition assay done to see how sEV internalization differs when IG2c Tyro3 domain is blocked.

-Figure 3 should have sEVs negative for Gas6 and Pros1 (negative control).

-Figure 4 should have antibody blocking assays/competition assay done to see how sEV internalization differs when the TAM receptors and/or MHC I are blocked and how it affects activation of CTLs. This should be done with the negative control of sEVs negative for Gas6 and Pros1 as well as sEVs with Gas6 and Pros1. These sEVs should be utilized for both in vitro and in vivo studies.

-Tumor progression data from figure 4F and 4G should be shown.

-Figure 4 the in vitro studies should be validated using more than one dendritic cell line.

-A figure describing the mechanism described in this manuscript of Tyro-3 mediated uptake of sEVs must be shown.

-It must be explicitly stated for the in vivo experiment that female mice were used in the figure legend. Similar studies must be done using male mice to ensure no sex differences as of figure 4F and 4G.

-The origin from which the cells used in this study came form must be described in detail including the sex.

**********

6. PLOS authors have the option to publish the peer review history of their article (what does this mean?). If published, this will include your full peer review and any attached files.

If you choose “no”, your identity will remain anonymous but your review may still be made public.

**Do you want your identity to be public for this peer review?**  For information about this choice, including consent withdrawal, please see our Privacy Policy.

Reviewer #1: No

Reviewer #2: **Yes:** Kevinn Eddy

**********

[NOTE: If reviewer comments were submitted as an attachment file, they will be attached to this email and accessible via the submission site. Please log into your account, locate the manuscript record, and check for the action link "View Attachments". If this link does not appear, there are no attachment files.]

To ensure your figures meet our technical requirements, please review our figure guidelines: https://journals.plos.org/plosone/s/figures

You may also use PLOS’s free figure tool, NAAS, to help you prepare publication quality figures: https://journals.plos.org/plosone/s/figures#loc-tools-for-figure-preparation.

NAAS will assess whether your figures meet our technical requirements by comparing each figure against our figure specifications.

# Author comment: Author response to Decision Letter 1

**DOI:** 10.1371/journal.pone.0355054.r002

30 Jun 2026

Suzie Chen

Academic Editor, PLOS One

RE: Ms No.: PONE-D-26-03793

Dear. Prof. Chen

We appreciate the Reviewer's comments for our manuscript entitled " Tyro3 facilitates cytoplasmic delivery of extracellular vesicle contents and antigen presentation via major histocompatibility

complex class I in dendritic cells"

(PONE-D-26-03793)

First of all, We would like to thank the Academic Editor and the two reviewers for their helpful comments. We have carefully considered all comments and revised the manuscript accordingly. We thus feel that the attached revised manuscript is complete in this regard. We provide point-by-point responses to the journal requirements, the Academic Editor’s comments, and the reviewers’ comments.

Point by point Reply for the Journal Requirements�

Requirement #1), PLOS ONE style requirements.

"Please ensure that your manuscript meets PLOS ONE's style requirements, including those for file naming."

We have revised the manuscript to comply with PLOS ONE's style requirements, including the requirements for file naming. The revised manuscript, the marked-up manuscript, and this response letter have been prepared and uploaded according to the journal's instructions.

Requirement #2), Original images for blot and gel data.

"PLOS ONE now requires that authors provide the original uncropped and unadjusted images underlying all blot or gel results reported in a submission’s figures or Supporting Information files."

We have provided the original uncropped and unadjusted images underlying the blot and phospholipid overlay data reported in the manuscript and Supporting Information files. These raw image data are included as Figure S8. We have also ensured that the revised figures comply with PLOS ONE’s requirements for blot/gel reporting and figure preparation. No raw blot/gel images are unavailable.

Requirement #3), Funding information.

"We note that the grant information you provided in the ‘Funding Information’ and ‘Financial Disclosure’ sections do not match. When you resubmit, please ensure that you provide the correct grant numbers for the awards you received for your study in the ‘Funding Information’ section."

We appreciate the editorial office’s careful check. We have resolved the discrepancy between the Funding Information and Financial Disclosure sections by correcting the grant information in both the submission system and the Funding Information section of the revised manuscript (Lines 689-697).

Requirement #4), Funding information.

"Please state what role the funders took in the study. If the funders had no role, please state: 'The funders had no role in study design, data collection and analysis, decision to publish, or preparation of the manuscript.'"

We have included the requested Role of Funder statement in the cover letter. The revised Funding information section of the manuscript also includes the following statement: "The funders had no role in study design, data collection and analysis, decision to publish, or preparation of the manuscript."

Requirement #5), Ethics statement.

"Please include your full ethics statement in the ‘Methods’ section of your manuscript file."

We have included a full ethics statement in the Methods section of the revised manuscript. The statement includes the full name of the animal experiment committee that approved the study, the approval number, and the animal care and euthanasia procedures. We also clarified that no human participants, primary human samples, or identifiable human data were used in this study and that informed consent was therefore not applicable.

Requirement #6), Citation of previously published works.

"If the reviewer comments include a recommendation to cite specific previously published works, please review and evaluate these publications to determine whether they are relevant and should be cited."

This comment is related to comment #2 from Reviewer 1. We have reviewed the previously published work recommended by the reviewer and evaluated its relevance to our study. Because Rothlin et al. (Nat Rev Immunol, 2015) provides important background on the roles of TAM receptors in immune regulation, we considered this work relevant and have cited it in the revised manuscript.

Additional Editor Comments:

"Please read the suggestions and comments from each reviewers and answer each comments specifically with details. The reviewers took a lot of time to review the manuscript please read their comments carefully and respond properly."

We thank the Academic Editor for this comment. We have carefully read all comments from the reviewers and have addressed each point in detail. Point-by-point responses to all reviewer comments are provided below, and the corresponding changes have been incorporated into the revised manuscript where appropriate.

Point by point Reply for the comments of Reviewer #1:

Comment #1) Insufficient information in the manuscript and figure legends.

"The manuscript generally lacks important information that is needed to understand it. Figure legends are usually too brief, and figures are difficult to understand. Key materials are not well-explained. Need an explanation for each material presented."

We agree that several key materials and experimental tools were not sufficiently explained in the original manuscript. We have therefore revised the manuscript and figure legends extensively. In the revised manuscript, we added a detailed description of the CD63-based sEV tools in the “Preparation of sEVs” section (Lines 548–553). We also clarified the purpose of each CD63-based sEV tool in the Results sections, including CD63-NLuc-sEVs (Lines 104–105), CD63-HiBiT-sEVs (Lines 221–224), CD63-dClover2-sEVs (Lines 233–235), and CD63-OVA-sEVs (Lines 311–314). We further defined +Gas6-sEVs and +Pros1-sEVs as sEVs preincubated with Gas6 or Pros1, respectively (Lines 119–122). We also substantially expanded the legends for Figures 1–4 (Lines 142-160, 201-219, 278-295, and 324-351) to include the experimental design, cell lines, assay methods, quantification procedures, fluorescence colors, scale bars, and sample numbers. We also revised the figure titles to better reflect the contents of each figure.

Comment #2), TAM receptor expression in DC2.4 cells.

" Please discuss or justify the absence of detectable TAM receptor expression in this dendritic cell line (DC2.4)."

In the revised Discussion, we now explicitly discuss this point (Line 393-405). As the reviewer noted, TAM receptors are important regulators of immune homeostasis in dendritic cells and other antigen-presenting cells. However, we did not detect endogenous surface expression of Tyro3, Axl, or Mertk in parental DC2.4 cells by flow cytometry under our assay conditions. Previous studies have shown that the expression patterns of individual TAM receptors vary depending on the DC subset, differentiation conditions, activation status, inflammatory context, and detection method. We also note that DC2.4 is an immortalized dendritic cell line and may not fully recapitulate the TAM receptor expression patterns of primary DC populations. Accordingly, we used DC2.4 cells individually expressing exogenous TAM receptors to evaluate the receptor-specific contribution of Tyro3, Axl, and Mertk to sEV association and internalization.

Comment #3) Rationale for the use of DC2.4 and HeLa cells.

"Rationale for switching between cell type: the study switches between DC2.4 cells and HeLa cells for different analyses, but the rationale for this choice is not clearly explained."

In the originally submitted manuscript, HeLa cells were used for confocal microscopy analyses because their flat morphology is suitable for obtaining clear intracellular images. However, we agree with the reviewer that the use of different cell types for different analyses was insufficiently justified and could make the interpretation of the results difficult. To address this concern, we repeated the HeLa-based confocal microscopy experiments using DC2.4-derived dendritic cell lines, which are the main cellular model used in this study. Accordingly, we replaced the previous HeLa cell data with new DC2.4 cell data in the revised manuscript. The revised data are now shown in Figure 3B and Figure 4E. We also revised the corresponding Results sections and figure legends to reflect these changes.

Comment #4), Visualization of sEV internalization in dendritic cells.

"Given the importance of EV uptake in antigen presentation in dendritic cells, visualization sEV internalization in dendritic cells would be informative."

We thank the reviewer for this helpful suggestion. In the revised manuscript, we visualized the intracellular localization of CD63-dClover2-sEV-derived signals in DC2.4 cells expressing Tyro3 or its ectodomain deletion mutants. The schematic illustration of the Tyro3 mutants, which was previously shown as Figure 2F, has been reorganized and is now shown as Figure 3A. The new confocal microscopy data obtained using DC2.4-derived cells are shown in Figure 3B. The corresponding Results section has been revised accordingly (Lines 268–276), and the figure legend has also been updated.

Point by point Reply for the comments of Reviewer #2:

Comment #1), Improvement of figure readability.

"Improvements in all the figures are required to improve their readability as it is blurry."

In the revised manuscript, we have improved the readability of all figures by increasing the font size and reorganizing the layout of the figure panels. We also prepared and uploaded higher-resolution figure files, within the file-size limits accepted by the PLOS ONE submission system.

Comment 2), Reorganization of sEV characterization data.

"Gas-6, Pros-1, and CD63 expressions in these sEVs in figure 2 should be present in figure 1 as chronologically it makes more sense after Figure 1B."

We thank the reviewer for this helpful suggestion. As suggested, we have reorganized the figures and moved the western blotting data for Gas6 and Pros1 in the sEV preparations from the original Figure 2A to the revised Figure 1D. This revision allows the characterization of the sEV preparations to be presented before the subsequent binding and cellular association assays. We have also revised the corresponding text in the Results section accordingly (Lines 114–119).

Comment 3), Evaluation of sEV preparation by electron microscopy.

"The purity of the isolation method of these sEV’s must be evaluated using electron micrograph."

We thank the reviewer for this important suggestion. In response, we performed transmission electron microscopy analysis of the sEV preparations isolated from Expi293F cells. The electron micrograph has been added as Figure 1C in the revised manuscript. We also revised the corresponding Results section to describe the TEM-based evaluation of the sEV preparations (Lines 112–114) and added the experimental details to the Materials and Methods section (Lines 565–567).

Comment #4), Pull-down assay.

"There should be figure 1 describing the readout of figure 1c to help the reader conceptualize the data."

As requested, we have added a schematic diagram of the pull-down assay workflow to the revised Figure 1E. This schematic illustrates how TAM receptor ectodomains were immobilized on beads, incubated with CD63-NLuc-sEVs, washed, and then evaluated by NanoLuc luciferase activity.

Comment #5), Background control in the pull-down assay.

"Figure 1c there should be a plain supernatant that has gone through the same process as the sEVs in figure 1 to evaluate the background signal that has no sEVs."

We thank the reviewer for this important suggestion. We agree that a processed no-sEV supernatant would be an appropriate control for estimating the absolute background signal in this assay. In the present experiment, NLuc activity was used as a readout for CD63-NLuc-labeled sEVs bound to individual TAM-ECDs. CD63-NLuc-sEVs, +Gas6-sEVs, and +Pros1-sEVs were applied as equivalent NLuc inputs and subjected to the same pull-down, washing, and detection procedures. Therefore, Fig 1F represents a relative comparison of CD63-NLuc-labeled sEV binding to individual TAM-ECDs under different ligand conditions. To avoid overinterpretation, we have clarified in the revised Results section that this assay was designed to evaluate relative TAM-ECD-associated CD63-NLuc-sEV signals under matched input conditions (Lines 123–127).

Comment #6), Sample sizes in figure legends.

"Sample sizes for all experiments must be explicitly stated in the figure legend."

In the revised manuscript, we have added the sample sizes to the relevant figure legends for Figure 1–4 (Lines 142-160, 201-219, 278-295, and 324-351).

Comment #7), Phosphatase treatment of phospholipids.

"For figure 1e and 1f phosphatase should be added to the phospholipids at varying concentrations to evaluate if the binding would be abrogated."

We thank the reviewer for this suggestion. In this study, we did not perform phosphatase treatment of the phospholipids. The phospholipid overlay assay was used to evaluate the relative binding of TAM ligands and TAM-ECDs to individual phospholipids under defined conditions. We agree that additional competition or blocking experiments, such as masking phosphatidylserine with annexin V, would further support the specificity of the interaction. We have noted this point as a limitation and an important subject for future study.

Comment #8), Use of HeLa cells in Figure 2.

"In figure 2, why weren’t the results of HeLa cells shown as it was mentioned that both DC2.4 and HeLa cells were used. The results of this must be shown."

We appreciate the reviewer’s careful reading. The experiments shown in Figure 2 were performed using DC2.4 cells, not HeLa cells. In the original manuscript, the sentence “We assessed the binding and uptake of sEVs by TAM-expressing cells using DC2.4 and HeLa cells” (original Lines 153–154) was misleading, as it could be interpreted to mean that the sEV association and uptake experiments were performed in both DC2.4 and HeLa cells. In the revised manuscript, we have corrected this description to clearly indicate that the Figure 2 experiments were performed using TAM receptor-expressing DC2.4 cells (Lines 180–182).

Comment #9), Validation in another dendritic cell line.

"All figure 2 data and data using one dendritic cell line must be validated in another dendritic cell line. The authors mentioned that they did similar experiments in HeLa cells; this must be shown."

We thank the reviewer for this important comment. We agree that validation in another dendritic cell line would provide additional support for the findings shown in Figure 2. However, in the present revision, we did not perform the full set of Figure 2 experiments in another dendritic cell line. Therefore, we have revised the manuscript to clearly state that the Figure 2 experiments were performed using TAM receptor-expressing DC2.4 cells and have limited our interpretation accordingly. We agree that validation in additional dendritic cell models remains an important future direction.

As also described in our response to Comment #8 from Reviewer 2, the original statement referring to both DC2.4 and HeLa cells was misleading. We have corrected this description in the revised manuscript.

Comment #10), Functional evaluation of Gas6-dependent sEV association and uptake.

"Figures 2B and 2C should have sEVs negative for Gas6 and Pros1 (negative control)."

We thank the reviewer for this important suggestion. We agree that Gas6/Pros1-negative sEVs would be a useful negative control. However, generating such sEVs from Expi293F producer cells would require additional genetic manipulation and careful validation to confirm that sEV production, size distribution, and CD63-reporter content are not substantially affected. Therefore, in the present revision, we performed a functional inhibition experiment using RU-302, a Gas6–TAM interaction inhibitor. Because the HiBiT-LgBiT system more directly evaluates sEV internalization than the CD63-NLuc-sEV system, we performed this additional experiment using CD63-HiBiT-sEVs and LgBiT-expressing DC2.4 cell lines. The new data are s

Attachment Submitted filename: Response to reviewers.docx

# Peer review: Decision Letter 1

**DOI:** 10.1371/journal.pone.0355054.r003

**Authors:** Suzie Chen

16 Jul 2026

Tyro3 facilitates cytoplasmic delivery of extracellular vesicle contents and antigen presentation via major histocompatibility complex class I in dendritic cells

PONE-D-26-03793R1

Dear Dr. Koyama,

We’re pleased to inform you that your manuscript has been judged scientifically suitable for publication and will be formally accepted for publication once it meets all outstanding technical requirements.

Within one week, you’ll receive an e-mail detailing the required amendments. When these have been addressed, you’ll receive a formal acceptance letter and your manuscript will be scheduled for publication.

An invoice will be generated when your article is formally accepted. Please note, if your institution has a publishing partnership with PLOS and your article meets the relevant criteria, all or part of your publication costs will be covered. Please make sure your user information is up-to-date by logging into Editorial Manager at Editorial Manager® and clicking the ‘Update My Information' link at the top of the page. For questions related to billing, please contact billing support.

If your institution or institutions have a press office, please notify them about your upcoming paper to help maximize its impact. If they’ll be preparing press materials, please inform our press team as soon as possible -- no later than 48 hours after receiving the formal acceptance. Your manuscript will remain under strict press embargo until 2 pm Eastern Time on the date of publication. For more information, please contact onepress@plos.org.

Kind regards,

Suzie Chen

Academic Editor

PLOS One

Additional Editor Comments (optional):

Reviewers' comments:

Reviewer's Responses to Questions

**Comments to the Author**

1. If the authors have adequately addressed your comments raised in a previous round of review and you feel that this manuscript is now acceptable for publication, you may indicate that here to bypass the “Comments to the Author” section, enter your conflict of interest statement in the “Confidential to Editor” section, and submit your "Accept" recommendation.

Reviewer #1: (No Response)

Reviewer #2: All comments have been addressed

**********

2. Is the manuscript technically sound, and do the data support the conclusions?

The manuscript must describe a technically sound piece of scientific research with data that supports the conclusions. Experiments must have been conducted rigorously, with appropriate controls, replication, and sample sizes. The conclusions must be drawn appropriately based on the data presented.

Reviewer #1: (No Response)

Reviewer #2: Yes

**********

3. Has the statistical analysis been performed appropriately and rigorously?

Reviewer #1: (No Response)

Reviewer #2: Yes

**********

4. Have the authors made all data underlying the findings in their manuscript fully available?

The PLOS Data policy requires authors to make all data underlying the findings described in their manuscript fully available without restriction, with rare exception (please refer to the Data Availability Statement in the manuscript PDF file). The data should be provided as part of the manuscript or its supporting information, or deposited to a public repository. For example, in addition to summary statistics, the data points behind means, medians and variance measures should be available. If there are restrictions on publicly sharing data—e.g. participant privacy or use of data from a third party—those must be specified.

Reviewer #1: (No Response)

Reviewer #2: Yes

**********

5. Is the manuscript presented in an intelligible fashion and written in standard English?

PLOS ONE does not copyedit accepted manuscripts, so the language in submitted articles must be clear, correct, and unambiguous. Any typographical or grammatical errors should be corrected at revision, so please note any specific errors here.

Reviewer #1: (No Response)

Reviewer #2: Yes

**********

6. Review Comments to the Author

Please use the space provided to explain your answers to the questions above. You may also include additional comments for the author, including concerns about dual publication, research ethics, or publication ethics. (Please upload your review as an attachment if it exceeds 20,000 characters)

Reviewer #1: (No Response)

Reviewer #2: Thank you for addressing all my comments. I strongly suggest that all responses to the reviewers must be addressed in the manuscript to ensure the readers know the limitations of the study.

**********

7. PLOS authors have the option to publish the peer review history of their article (what does this mean?). If published, this will include your full peer review and any attached files.

If you choose “no”, your identity will remain anonymous but your review may still be made public.

**Do you want your identity to be public for this peer review?**  For information about this choice, including consent withdrawal, please see our Privacy Policy.

Reviewer #1: No

Reviewer #2: **Yes:** Kevinn Eddy

**********

# Editor report: Acceptance letter

**DOI:** 10.1371/journal.pone.0355054.r004

**Authors:** Suzie Chen

PONE-D-26-03793R1

PLOS One

Dear Dr. Koyama,

I'm pleased to inform you that your manuscript has been deemed suitable for publication in PLOS One. Congratulations! Your manuscript is now being handed over to our production team.

At this stage, our production department will prepare your paper for publication. This includes ensuring the following:

* All references, tables, and figures are properly cited

* All relevant supporting information is included in the manuscript submission,

* There are no issues that prevent the paper from being properly typeset

You will receive further instructions from the production team, including instructions on how to review your proof when it is ready. Please keep in mind that we are working through a large volume of accepted articles, so please give us a few days to review your paper and let you know the next and final steps.

Lastly, if your institution or institutions have a press office, please let them know about your upcoming paper now to help maximize its impact. If they'll be preparing press materials, please inform our press team within the next 48 hours. Your manuscript will remain under strict press embargo until 2 pm Eastern Time on the date of publication. For more information, please contact onepress@plos.org.

You will receive an invoice from PLOS for your publication fee after your manuscript has reached the completed accept phase. If you receive an email requesting payment before acceptance or for any other service, this may be a phishing scheme. Learn how to identify phishing emails and protect your accounts at https://explore.plos.org/phishing.

If we can help with anything else, please email us at customercare@plos.org.

Thank you for submitting your work to PLOS ONE and supporting open access.

Kind regards,

PLOS ONE Editorial Office Staff

on behalf of

Dr. Suzie Chen

Academic Editor

PLOS One
